# CRISPR/Cas9 and *Agrobacterium tumefaciens* virulence proteins synergistically increase efficiency of precise genome editing via homology directed repair in plants

**DOI:** 10.1093/jxb/erad096

**Published:** 2023-03-15

**Authors:** Ye Tang, Zhennan Zhang, Zhiyuan Yang, Jiahe Wu

**Affiliations:** State Key Laboratory of Plant Genomics, Institute of Microbiology, Chinese Academy of Sciences, Beijing, China; State Key Laboratory of Plant Genomics, Institute of Microbiology, Chinese Academy of Sciences, Beijing, China; State Key Laboratory of Plant Genomics, Institute of Microbiology, Chinese Academy of Sciences, Beijing, China; State Key Laboratory of Plant Genomics, Institute of Microbiology, Chinese Academy of Sciences, Beijing, China; Instituto de Agrobiotecnologia del Litoral, Argentina

**Keywords:** *Agrobacterium tumefaciens* virulence proteins, CRISPR/Cas9, DNA linker, double-strand breaks (DSBs), homology directed repair (HDR), precise genome editing, T-DNA donor

## Abstract

CRISPR/Cas9 genome editing and *Agrobacterium tumefaciens*-mediated genetic transformation are widely-used plant biotechnology tools derived from bacterial immunity-related systems, each involving DNA modification. The Cas9 endonuclease introduces DNA double-strand breaks (DSBs), and the *A. tumefaciens* T-DNA is released by the VirD2 endonuclease assisted by VirDl and attached by VirE2, transferred to the plant nucleus and integrated into the genome. Here, we explored the potential for synergy between the two systems and found that Cas9 and three virulence (Vir) proteins achieve precise genome editing via the homology directed repair (HDR) pathway in tobacco and rice plants. Compared with Cas9T (Cas9, VirD1, VirE2) and CvD (Cas9-VirD2) systems, the HDR frequencies of a foreign *GFPm* gene in the CvDT system (Cas9-VirD2, VirD1, VirE2) increased 52-fold and 22-fold, respectively. Further optimization of the CvDT process with a donor linker (CvDTL) achieved a remarkable increase in the efficiency of HDR-mediated genome editing. Additionally, the HDR efficiency of the three rice endogenous genes *ACETOLACTATE SYNTHASE* (*ALS*), *PHYTOENE DESATURASE* (*PDS*), and *NITROGEN TRANSPORTER 1.1 B* (*NRT1.1B*) increased 24-, 32- and 16-fold, respectively, in the CvDTL system, compared with corresponding Cas9TL (Cas9T process with a donor linker). Our results suggest that collaboration between CRISPR/Cas9 and *Agrobacterium*-mediated genetic transformation can make great progress towards highly efficient and precise genome editing via the HDR pathway.

## Teaser

CRISPR/Cas9 and *Agrobacterium tumefaciens*virulence proteins synergistically facilitate the precise delivery of a well-protected donor template to the induced double strand-break site, and achieve genome editing via homology directed repair.

## Introduction

The clustered regularly interspaced short-palindromic repeat (CRISPR) system is a mechanism of adaptive immunity in bacteria and archaea against invading viruses and plasmids (Jinek *et al.*, 2012). Two classes of the CRISPR system, Class 1 and Class 2, have currently been identified (Makarova et al., 2017a, b). Owing to the modular feature, CRISPR-Cas9 belongs to the Class 2 (type II) CRISPR system, and is the most widely used system for genome editing in eukaryotes (Makarova et al., 2015, 2020; Wright *et al.*, 2016;Koonin *et al.*, 2017 ). Over the past decade, the development of the CRISPR/Cas9 genome editing system has revolutionized plant biotechnology by allowing the efficient introduction of targeted mutations in any gene within a single generation (Gao, 2021), including the targeting of multiple genes simultaneously (Armario Najera *et al.*, 2019). The CRISPR-Cas9 system contains two main elements: Cas9 DNA endonuclease and a single guide RNA (sgRNA). The sgRNA exerts a complementary 20-nucleotide sequence of the target DNA (called the protospacer), which is directly next to a protospacer adjacent motif (PAM) recognized by Cas9 (Collias and Beisel, 2021). *Streptococcus pyogenes* Cas9 is the most highly used endonuclease to introduce DNA double-strand breaks (DSBs) at the sites defined by sgRNA before PAM (Zhang *et al.*, 2019). DSBs are typically repaired by the endogenous non-homozygous end joining (NHEJ) pathway, which is error prone and results mostly in small indels at the target site, or less frequently in larger indels or rearrangements (Hsu *et al.*, 2014;Bortesi *et al.*, 2016; Xue and Greene, 2021). However, the provision of a donor repair template homozygous to the region around the break site allows repair via the alternative homology-directed repair (HDR) pathway, which can be used for targeted deletions, insertions (knock-in) or substitutions, including single-nucleotide replacements (Komor *et al.*, 2016; Chang *et al.*, 2017;Jayavaradhan *et al.*, 2019; G.L. Li *et al.*, 2020;Zhang *et al.*, 2020). The efficiency of HDR is generally much lower than NHEJ in the repair of DSBs. Thus, many researchers have attempted to increase HDR efficiency either by creating an environment that favours HDR over NHEJ, or by targeting the genome repair template more effectively (Gil-Humanes *et al.*, 2017;Nambiar *et al.*, 2019;Rees *et al.*, 2019;Wienert *et al.*, 2020;Wen *et al.*, 2021). For example, to improve the efficiency of HDR, regulated cell cycle progression or temporal control of Cas9 expression limit the DNA nuclease activities to the phase when HDR is naturally active (Gutschner *et al.*, 2016). Several small molecules are used to inhibit NHEJ or up-regulate HDR efficiency during DSB repairs (Li *et al.*, 2017;Chen *et al.*, 2018;Lee *et al.*, 2022). Furthermore, increasing the concentration of the DNA donor template, carrying the RNA template into nuclei, or chemically modifying the oligonucleotide to stabilize the templates, can significantly increase HDR efficiency compared with the low concentration or unprotected template control (Gutierrez-Triana *et al.*, 2018;Li *et al.*, 2019).

Recently, HDR-mediated genome editing was found to be significantly more efficient compared with the control, when the donor repair template that was naked or only end-modified was attached to the editing complex during transfer to the organism DSB sites; this increased the template stabilization/concentration in the DSBs by covalently binding to endonuclease or a tag protein (Aird *et al.*, 2018; Savic *et al.*, 2018;Ali *et al.*, 2020). For example, Porcine Circovirus 2 (PCV) Rep protein, a histidine-hydrophobic residue-histidine (HUH) endonuclease, was fused to Cas9 and could tether the unmodified donor DNA template to form the stable covalent RNP-ssODN complex, by which up to a 30-fold enhanced HDR efficiency was realized, compared with Cas9 (Lovendahl *et al.*, 2017;Aird *et al.*, 2018). Another endonuclease, VirD2 relaxase, covalently bound to the DNA repair template (DRT), was fused to Cas9, which enhanced the precise replacement ratio by 5- to 6-fold, compared with Cas9 alone (Ali *et al.*, 2020). Furthermore, the SNAP-tag protein fused to Cas9 covalently links the donor template into the proximity of the DSBs, resulting in increase of the HDR efficiency ratios up to 24-fold compared with Cas9 alone (Savic *et al.*, 2018). However, novel approaches on the precise delivery of a sufficient amount of repair-template to the DSB site remain to be explored to increase the efficiency of HDR in plants and animals.

*Agrobacterium*, a soilborne phytopathogen, possesses an efficient and precise DNA delivery system for plants, which also is the only known example for inter-kingdom DNA transfer in organism (De Saeger *et al.*, 2021). Now, *Agrobacterium*-mediated genetic transformation technology has become the most widely used biotechnology tool in plants, yeast, fungi, and even animals (Ziemienowicz *et al.*, 1999;Krenek *et al.*, 2015;Song *et al.*, 2015;Li and Pan, 2017;Hooykaas *et al.*, 2018). *Agrobacterium* virulence (Vir) proteins encoded on the tumour-inducing (Ti) plasmid are required for T-DNA transfer and integration into the plant genome (Gordon and Christie, 2014). Specifically, VirD2 recognizes a 25 bp imperfect direct repeat sequence at the T-DNA border and cleaves the conserved sequences with the assistance of the topoisomerase VirD1, leaving VirD2 tightly attached to the 5ʹ end of the nicked T-DNA (Relić *et al.*, 1998;Kralemann *et al.*, 2022). The exposed T-DNA tail is quickly coated with single-stranded DNA (ssDNA) binding protein VirE2, protecting it from endogenous nucleases (X. Li *et al.*, 2020). T-DNA, VirD2 and VirE2 form a T-DNA-protein complex, which enters into the plant nucleus, and then the T-DNA is finally integrated into the genome (Gelvin, 2017). Therefore, collaboration between these Vir proteins provides an exquisite mechanism by which foreign DNA can be well protected and transferred into plant cells and enter into the nucleus.

**Fig. 1.**
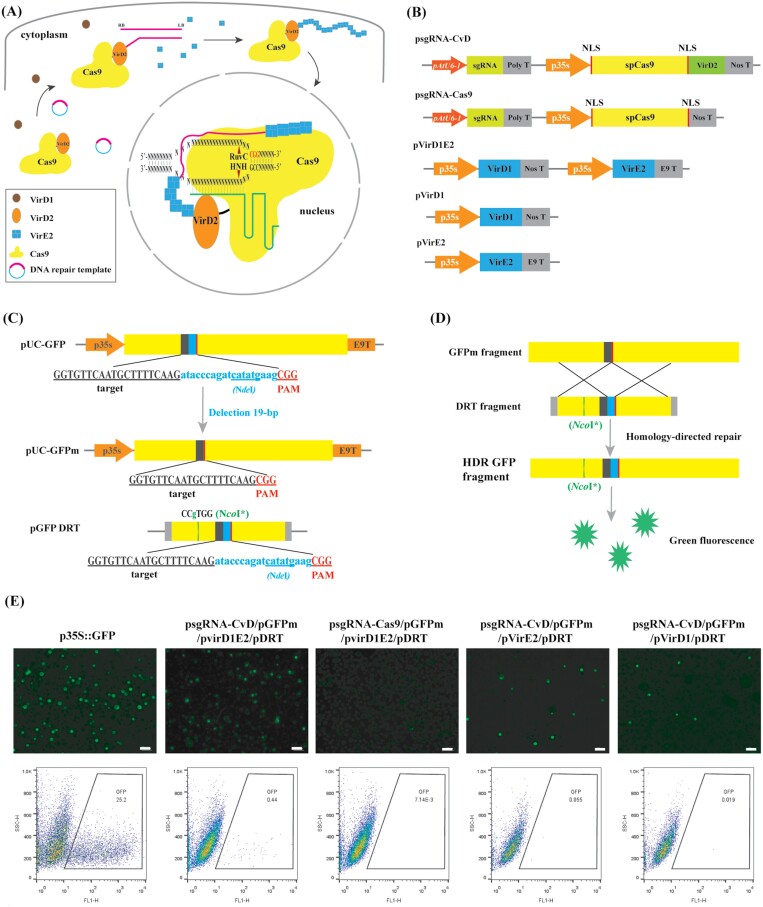
CRISPR/Cas9 and *Agrobacterium tumefaciens* virulence proteins act synergistically during genome editing in tobacco protoplasts. (A) Schematic diagram of CRISPR/Cas9 and *Agrobacterium tumefaciens* virulence proteins VirD1, VirD2, and VirE2 acting synergistically to achieve precise genome editing in plants. In the cytoplasm, the fused protein Cas9-VirD2 (CvD) cleaves the conserved sequences of DNA repair template (DRT) with the assistance of the VirD1. Next, the exposed DNA tail is quickly coated with VirE2. Finally, the CvD fused protein and covalently bound well-protected DRT move into the nucleus. (B) Structure of the psgRNA-CvD, psgRNA-Cas9, pvirD1E2, pVirD1, and pVirE2 vectors. (C) Structure of the pUC-GFP, pUC-GFPm, and pGFP-DRT vectors. Structure of the DRT; the grey bars are the LB and RB sequences. The sgRNA target sequences (black box) are shown below the construct, the PAM in a red box, and the 19 nt deletion in a blue box, with blue lowercase letters containing the *Nde*I site underlined in blue. The *Nco*I* represents *Nco*I with a synonymous (no amino acid) mutation, to abolish the original site, shown in green lowercase letters and green box. (D) Schematic of GFPm and the repair template (GFP DRT) to restore GFP activity. (E) The abundance of GFP^+^ tobacco protoplasts as determined by microscopy (upper row, scale bar=100 µm) and flow cytometry (lower row).

In this study, we consider the *A. tumefaciens* virulence proteins that carry T-DNA into the nucleus and protect it from degradation; therefore, union of the CRISPR/Cas9 and *A. tumefaciens* genetic transformation system can be expected to synergistically increase efficiency of precise genome editing via HDR. Thus, we propose that a Cas9-VirD2 fusion protein (CvD), VirD1, and VirE2 can facilitate the precise delivery of a well-protected donor template to the Cas9-induced DSB site, to increase HDR ratio (Fig. 1A).

## Materials and methods

### Construction of CRISPR/Cas9 and Vir protein expression vectors

The *Streptococcus pyogenes Cas9* gene and *Agrobacterium tumefaciens VirD1*, *VirD2* and *VirE2* genes were codon optimized for plants and synthesized by Tsingke (Beijing, China). The fused *Cas9-VirD2* gene (*CvD*) was transferred to vector pUC57 (Tsingke, Beijing, China) flanked by the CaMV35S promoter and NOS terminator. The sgRNA fragment was flanked by the *Arabidopsis thaliana* U6-1*/ Oryza sativa* U6c promoter upstream, and the poly-T terminator downstream. The sgRNA and CvD cassettes were placed in tandem on vector psgRNA-CvD. The *CvD* gene in psgRNA-CvD was replaced with the *Cas9* gene to generate vector psgRNA-Cas9. The *VirE2* gene was transferred to vector pUC flanked by the CaMV35S promoter and E9 terminator to generate vector pVirE2. The *VirD1* gene was introduced into binary vector pBI121 (Tsingke, Beijing, China) by replacing the *β-glucuronidase* (*gusA)* gene coding sequence to generate vector pVirD1. The 35S-VirD1-NOS expression cassette was amplified using primers containing *Eco*RI restriction sites, and the resulting product was transferred to vector pVirE2 (linearized with *Eco*RI) to generate tandem vector pVirD1E1 containing constructs 35S-VirD1-NOS and 35S-VirE2-E9. The vectors were constructed using the Trelief SoSoo Cloning Kit (Beijing, China) and all primers are listed in Supplementary Table S1.

### Construction of the GFPm reporter vector and repair template

The *GFPm* gene was generated by removing a 19 bp segment (219–237 bp) from the functional *GFP* gene. We designed two pairs of primers (GFPm-F1/R1 and GFPm-F2/R2) to amplify the 5ʹ and 3ʹ segments, and the resulting amplicons were linked by PCR with the outermost primers to create the *GFPm* sequence. This was used to replace the *GFP* gene (flanked by the CaMV35S promoter and E9 terminator) in vector pUC-GFP (Tang *et al.*, 2019) following digestion with *Kpn*I and *Spe*I, yielding vector pUC-GFPm. The repair template (GFP DRT) was chemically synthesized, including the missing 19 bp sequence with a *Nde*I site, another mutation to eliminate the *Nco*I site, and a recognition sequence for VirD2. This fragment was inserted into pClone007 (Tsingke, Beijing, China) to generate pGFP DRT. An 80 bp sequence from the *gusA* gene was amplified from pBI121 as a DNA linker, and was placed between the VirD2 recognition sequence and the homology region spanning the DSB to form GFP linker DRT, which was inserted into pClone007 to generate the GFP linker DRT vector. DRTs containing an 80 bp linker were also chemically synthesized for three endogenous rice genes [*ACETOLACTATE SYNTHASE* (*ALS*), *PHYTOENE DESATURASE* (*PDS*), and *NITROGEN TRANSPORTER 1.1 B* (*NRT1.1B*)] and transferred to pClone007 to generate the vectors pDRT-OsALS, pDRT-OsPDS, and pDRT-OsNRT1.1B, respectively. The *OsALS* HDR construct with A96V mutation from DRT-OsALS fragment confers rice plant resistance to the herbicide imazamox. The *GFPm* gene mentioned above was introduced into pBI121 to generate vector pBI121-GFPm for *Agrobacterium tumefaciens*-mediated genetic transformation in tobacco. The vectors were constructed using the Trelief SoSoo Cloning Kit, and all primers are listed in Supplementary Table S1.

### Protoplast isolation

Protoplasts were isolated from *Nicotiana benthamiana* and the japonica rice cultivar ‘Nipponbare’. *N. benthamiana* and rice seeds were cultivated in a sterilized soil mixture (a complex of organic matter soil: vermiculite = 3:1) in a growth chamber under 25 °C with a 16 h/8 h light/dark photoperiod, 400 µmol m^–2^ s^–1^and 75% relative humidity. Three-week-old tobacco mesophyll and rice sheath seedlings protoplasts were isolated as described previously (Shan *et al.*, 2013;Lei *et al.*, 2014) with slight modifications. Briefly, healthy fresh tobacco leaves (second, third and fifth true leaves) or young, non-wilting rice sheaths were cut into fine strips and soaked in 0.6 M mannitol for 10 min in the dark. The tobacco strips were then digested with enzymes (1.5% Cellulase R10, 0.75% Macerozyme R10) in 0.6 M mannitol, 10 mM MES (pH 5.7), 10 mM CaCl_2_ and 0.1% bovine serum albumin (BSA) at 25 °C for 3–4 h in the dark, shaking at 60 rpm. Rice strips were infiltrated with the same enzyme mix under vacuum in the dark for 30 min, and were incubated as above for 6–7 h. The digestion reaction was stopped by adding the same amount of W5 buffer (154 mM NaCl, 125 mM CaCl_2_, 5 mM KCl and 0.03% MES, pH 5.8). Protoplasts were collected by centrifugation at 100–300 × *g* to obtain pellets, which were washed twice with W5 buffer before resuspension in MMG buffer (15 mM MgCl_2_, 0.4 M mannitol and 0.1% MES, pH 5.8) to a final concentration of 2 × 10^7^ ml^–1^.

### PEG-mediated protoplast transformation

Protoplasts were transformed by incubation in 40% (w/v) PEG 4000, 0.2 M mannitol, and 0.1 M CaCl_2_. Combinations of plasmids were mixed at an equimolar ratio and added to 200 μl of the protoplast suspension. We then added 220 μl of the PEG solution and mixed gently but thoroughly for 15 min (tobacco) or 20 min (rice) in the dark. After incubation, the transformed protoplasts were harvested and resuspended in W5 solution, and then incubated at 23 °C for ~48 h under a light intensity of 50 μmol m^–2^ s^–1^. The protoplasts were then recovered for microscopy, flow cytometry or DNA extraction, as required.

### *Agrobacterium*-mediated gene transformation in tobacco

*Agrobacterium*-mediated genetic transformation in tobacco was performed as described previously (Niedbała *et al.*, 2021) with phytohormone content modifications. Briefly, tobacco leaf disk explants were used for transformation by the *A. tumefaciens* strain LBA4404 containing the binary expression vector pBI121-GFPm. The inoculated leaf disk explants were co-cultivated in Murashige and Skoog (MS) medium supplemented with 0.1 mg l^–1^ 1-Naphthaleneacetic acid (NAA) + 1.5 mg l^–1^ 6-benzylaminopurine (BAP) for 48 h in the dark. The explants were then transferred to the selective MS medium containing 0.1 mg l^–1^ NAA, 1.5 mg l^–1^ 6-BAP, 300 mg ^–1^ kanamycin, and 100 mg l^–1^ timentin for shoot regeneration.

### Callus induction in rice

Plant seeds from the current generation rice cultivar ‘Nipponbare’ were collected for callus induction. The cultivation conditions were the same as those of ‘Nipponbare’ mentioned above. The collected seeds were dehulled and the embryos were surface sterilized by dipping in 70% ethanol for 30 s, followed by incubation in 2% NaClO for 30 min. After rinsing three times in sterile water, the embryos were dried and placed on callus induction medium (4.1 g N6B5 salt (Phytotech, USA), 2 ml of 1 mg ml^–1^ 2,4-D, 0.5 g glutamine, 0.1 g inositol, 2.8 g proline, 0.5 g N-Z-Amine A, and 30 g sucrose to 1 litre distilled water, pH 5.8). After 1 week in the dark, the cultures were transferred to fresh induction medium and incubated in the dark to promote callus formation. The callus tissue was sub-cultured every 2 weeks and embryogenic callus was used for particle bombardment.

### Particle bombardment in rice

Embryogenic rice callus (6–9 weeks old) was transformed by particle bombardment as described previously (Liang *et al.*, 2018). Given that the HDR efficiency may largely depend on the amount of donor used, the CRISPR plasmid and *A. tumefaciens* vir protein plasmids were mixed with the donor plasmid in a molar ratio of 1:3, precipitated onto 0.6 mm gold particles, and co-transformed into rice immature embryogenic calli by particle bombardment, as described in Nguyen *et al.* (2022). We placed 60–80 callus pieces on a plate containing osmotic medium (4.43 g MS salt, 5 ml of 1 mg ml^–1^ 2,4-D, 90 g mannitol, and 30 g sucrose to 1 l distilled water, pH 6.0) for 3–4 h, before particle bombardment using the PDS1000/He system (Bio-Rad, Hercules, CA, USA) with a target distance of 6.0 cm from the stopping plate, and a helium pressure of 1100 psi. Each plate containing calli was bombarded twice, and the callus was incubated on osmotic medium in the dark overnight. The callus material was then transferred to induction medium for recovery for 1 week in the dark before transfer to recovery medium supplemented with 50 mg l^–1^ hygromycin for two rounds of selection in the dark, each lasting 5–6 weeks. Hygromycin-resistant callus tissue was used for DNA extraction, or was transferred to regeneration medium (4.1 g N6B5 salt, 1 ml of 2 mg ml^–1^ 6-BA, 8 μl of 25 mg ml^–1^ kinetin, 0.1 g inositol, 20 g sorbitol, 0.5 g N-Z-Amine A, and 30 g sucrose to 1 litre distilled water, pH 5.72), and then rooting medium (4.43 g MS salt, 0.1 g inositol, and 30 g sucrose to 1 litre distilled water, pH 5.8), each supplemented with 30 mg l^–1^ hygromycin, to regenerate plantlets. Regeneration was performed at 28 °C with a 16 h photoperiod at a light intensity of 400 μmol m^–2^ s^–1^ and 80% relative humidity.

### Analysis of herbicide tolerance

Bombarded callus was recovered as described above, but was selected on recovery medium supplemented with 50 mg l^–1^ hygromycin and 70 mg l^–1^ imazamox to confirm the presence of the A96V mutation, as described in Shimatani *et al.* (2018). To analyse herbicide tolerance in regenerated plants, we applied 0.07 mg l^–1^ imazamox to the leaves and monitored the plants for signs of toxicity.

### Flow cytometry

Protoplasts were sorted in a BD FACS Calibur device (BD Biosciences, Franklin Lakes, NJ, USA) fitted with a 100 μm nozzle to optimize survival. Gates were set to separate and enrich GFP^–^ and GFP^+^ protoplasts based on ~10000 events per experiment. Data were processed using FlowJo v10 (FlowJo, Ashland, OR, USA). The percentage of efficiency of HDR in flow cytometry analysis was calculated using the percentage of fluorescent cells over the events falling within the total protoplast sorting gate.

### Multi-cycle nested PCR

The accuracy of GFP HDR events was determined by multi-cycle nested PCR coupled with *Nco*I digestion, using genomic DNA extracted from pooled tobacco protoplasts by Trelief^TM^ Hi-Pure Plant DNA extracted kit (Tsingke, Beijing, China). Three specific primer pairs (35S/E9T, F1/R1 and F2/R2, listed in Supplementary Table S1) were designed for three rounds of nested PCR. In the first round, products amplified with primers 35S and E9T were digested with *Nco*I to enrich for GFP HDR fragments. These recovered fragments were used as templates in the second round (primers F1 and R1) and the products were again digested with *Nco*I to further enrich for GFP HDR fragments. The process was repeated for the third round (primers F2 and R2) and the major GFP HDR fragments after digestion with *Nco*I were purified by agarose gel electrophoresis and used as a template for the fourth round of PCR (a direct repeat of the third round). The resulting 321 bp amplicons were cleaved by *Nde*I into 234 bp and 87 bp fragments. All primers are listed in Supplementary Table S1.

### PCR restriction enzyme digestion assay (PCR/RE assay) and Sanger sequencing

Genomic DNA was amplified using a high-fidelity DNA polymerase (TSINGKE TP001 I-5™ 2×High-Fidelity Master Mix, Beijing, China) and corresponding gene-specific primers. The products were then digested with diagnostic restriction enzymes, and the fragments were separated by agarose gel electrophoresis to detect HDR events. The fragments were also sequenced using the Sanger method to identify the specific mutations.

### Deep amplicon sequencing

Genomic DNA was amplified in a first-round of PCR using gene-specific primers with a bridge sequence. The first-round PCR products were used as templates for the second-round PCR using barcode-specific primers. The second-round PCR products from different samples were pooled in equimolar amounts as a library for deep amplicon sequencing. The samples were sequenced commercially using the BGISEQ PE150 or BGISEQ PE250 platforms (BGI, Shenzhen, China). The percentage of efficiency HDR was calculated by the number of the targeting events over the total reads. All primers are listed in Supplementary Table S1.

### Digital droplet PCR (ddPCR)

Quantitative measurement of the gene editing frequency was conducted by ddPCR with site-specific primers and probes, as described previously (Beying *et al.*, 2020). Total DNA was extracted from protoplasts or callus tissue of *OsPDS* HDR and wild-type sample, and amplified using the primers GSP1 and GSP2, which were designed to amplify across the *OsPDS* locus. The probe labelled with 5ʹ-FAM was used to detect the original *OsPDS* sequence, and the probe labelled with 5ʹ-VIC was used to detect the HDR allele. The probes were conjugated to Iowa Black Hole Quencher 1 before the PCR. The primer and probe sequences were designed using Primer Premier v5.0 and were chemically synthesized by Sangon Biotech (Shanghai, China). For each sample, the ddPCR cocktail comprised 12 μl of 2 × ddPCR Supermix for Probes (no dUTP), 2 μl of each primer (10 μM), 0.6 μl of each probe (10 μM), 25 ng genomic DNA, and water to a final volume of 24 μl. Following this, 20 μl of the reaction mix and 70 μl of droplet generation oil were transferred into each hole of the droplet generation cartridge. The ddPCR was performed on a QX200 AutoDG Droplet Digital PCR system (Bio-Rad, America) and the samples were transferred to a QX200 droplet reader for analysis. All primers are listed in Supplementary Table S1.

### Statistical analysis

All data analysis and production of graphs were conducted with GraphPad Prism. The statistical tests were performed using SPSS. Differences in HDR ratio were determined by Student’s *t*-tests in this study. A significant level of 0.05 and 0.01 were used for statistical analysis.

## Results

### HDR efficiency evaluation in foreign *GFP* editing through combination of Cas9 and Vir proteins

To evaluate if CRISPR/Cas9 and *Agrobacterium tumefaciens* virulence proteins act synergistically and efficiently in genome editing via the HDR pathway (Fig. 1A), we generated a series of plant expression vectors, including psgRNA-CvD, psgRNA-Cas9, pVirD1E1, pVirD1, and pVirE2. The ­construct maps are shown in Fig. 1B with additional details in Supplementary Datasets S1-S3. The efficiency of HDR was determined by preparing a construct (pUC-GFPm) expressing an inactive green fluorescent protein gene (*GFPm*) under the control of the CaMV35S promoter, and a corresponding donor repair template plasmid (pGFP DRT). The *GFPm* gene featured an *Nco*I restriction site and a 19 bp deletion (from 219–237 bp) that generated a new sgRNA protospacer and PAM at the junction (Fig. 1C; Supplementary Fig. S1A; Supplementary Table S2). The DRT contained the missing 19 bp sequence (with an internal *Nde*I site), a point mutation to eliminate the *Nco*I site, and a recognition sequence for VirD2 (Fig. 1C, D; Supplementary Fig. S1B). We predicted that the HDR between *GFPm* and the DRT should restore GFP activity, and the *GFPm* and repaired *GFP* sequences distinguished by restriction analysis with *Nco*I and *Nde*I (Fig. 1C, D). PEG-mediated co-transformation of tobacco protoplasts with the HDR vectors, as indicated in Fig. 1B and Supplementary Table S2, was compared with a functional *GFP* gene driven by the CaMV35S promoter as a positive control. Flow cytometry analysis revealed that the positive control generated 25.2% GFP^+^ tobacco cells, whereas the HDR experiments did indeed lower the ratios of GFP^+^ tobacco cells by <1% (Fig. 1E; Supplementary Fig. S2). The transformed efficiency of GFP^+^ cells in positive control in this study was lower than those in previous reports (Shen *et al.*, 2014;Petersen *et al.*, 2019), possibly due to the different methods of tobacco transformation in various laboratories. However, the HDR experiments using the CvDT (CvD, VirD1 and VirE2) system achieved an efficiency of 4.4 × 10^–3^ compared with 7.1 × 10^–5^ for the equivalent process using Cas9T (Cas9, VirD1 and VirE2), indicating that the Cas9 fused with VirD2 achieves increased efficiency in HDR-mediated genome editing (Fig. 1E; Supplementary Fig. S2; Supplementary Dataset S1). To confirm that all elements in the CvDT editing system worked well together, we explored the individual roles of VirD1 and VirE2 in this system. We found that even in the presence of CvD fused protein, the efficiency of HDR was strongly reduced by the absence of VirD1 (5.5 × 10^–4^) or VirE2 (1.9 × 10^–4^), indicating that both VirD1 and VirE2 played vital roles in CvDT (Fig. 1E; Supplementary Fig. S2; Supplementary Dataset S1). Therefore, we performed a comparative experiment to test VirD1 and VirE2 function in the CvDT editing system with the only CvD-containing gene editing system previously reported by Ali *et al.* (2020). When both VirD1 and VirE2 were added, the efficiency of HDR remarkably increased in the CvDT system (4.0 × 10^–3^) compared with the gene editing system with only CvD present (1.3 × 10^–4^), suggesting that the two *A. tumefaciens* virulent proteins can increase the frequency of HDR events (Supplementary Fig. S3).

**Fig. 2.**
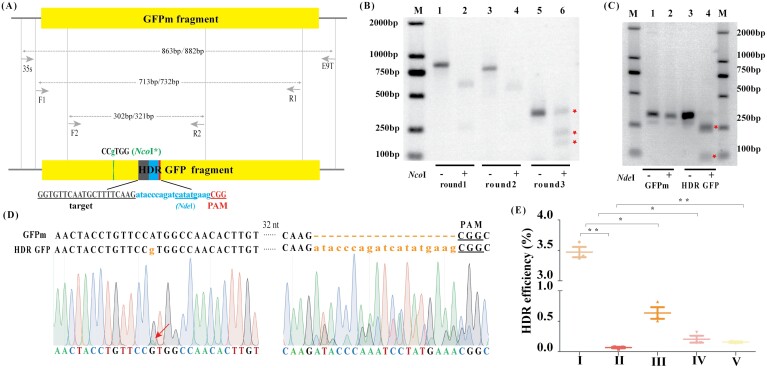
The detection of HDR events by multi-cycle nested PCR, Sanger and deep amplicon sequencing. (A) Schematic map of the HDR constructs, primer sets (35S/E9T, F1/R1 and F2/R2) and PCR amplicon sizes obtained from pUC-GFPm (values in bp before slash) and HDR GFP (values after slash). (B) GFP fragments in three rounds of nested PCR coupled with *Nco*I digestion. The red asterisk shows the third round of *Nco*I digestion products. Lanes 1, 3, and 5 represent three fragments (gradually becoming smaller) amplified by three rounds of nested PCR with primer sets 35S/E9T, F1/R1 and F2/R2, respectively; lanes 2, 4, and 6 show the corresponding amplicons digested with *Nco*I to yield products of 605 and 258 bp, 499 and 214 bp, and 170 and 132 bp, respectively. Successful HDR events generate *Nco*I-resistant amplicons. M: DL2000 Maker. (C) The *Nde*I digestion of PCR products generated using primer set F2/R2. Successful HDR events generate two bands following *Nde*I digestion. Lanes 1 and 2 show the products of plasmid GFPm (amplified with F2/R2) before and after digestion with *Nde*I (no change); lanes 3 and 4 show the fourth round of PCR from panel (B), amplified with F2/R2, before and after digestion with *Nde*I, resulting in 234 and 87 bp fragments, respectively. M: DL2000 Maker. (D) Sanger sequencing chromatogram of the third-round PCR products (shown in 2B) confirming the presence of HDR events. (E) Co-transformation of tobacco protoplasts with four combinations of plasmids carrying different genes and the repair template DRT and the HDR efficiency revealed by deep amplicon sequencing. I: GFPm+DRT+CvD+VirD1+VirE2; II: GFPm+DRT+Cas9+VirD1+VirE2; III: GFPm+DRT+CvD+VirE2; IV: GFPm+DRT+CvD+VirD1; V: GFPm+DRT+CvD. Values are means ±SD; n=3. Asterisks indicate significant differences (Student’s *t*-test, *P<0.05; **P<0.01).

The accuracy of the HDR events was evaluated by a combination of restriction digestion, PCR, and sequencing. Based on flow cytometry and fluorescence detection analysis, tobacco cells from the HDR experiments using CvDT were sampled to extract genomic DNA. The amplicons generated by multi-cycle nested PCR combined with *Nco*I digestion were cleaved by *Nde*I following successful HDR, whereas the corresponding *GFPm* amplicon was not (Fig. 2A-C; Supplementary Fig. S4). The precise boundaries of the HDR events were confirmed by Sanger sequencing (Fig. 2D). Deep amplicon sequencing supported the flow cytometry results with a similar trend (Fig. 2E; Supplementary Table S3). HDR experiments using the CvDT system achieved an efficiency of 3.5 × 10^–2^ based on the analysis of ~300 000 reads, compared with the Cas9T system (6.6 × 10^–4^), showing a 52-fold increase. Furthermore, HDR experiments using CvDT systems free of VirD1, VirE2, or both, attained 6.3 × 10^–3^, 2.0 × 10^–3^ and 1.5 × 10^–3^, respectively, which was significantly reduced, compared with the CvDT system. These data confirmed that VirD1 and VirE2 are able to increase HDR efficiency (Fig. 2E; Supplementary Table S3).

To verify whether the HDR takes place in the nucleus, we generated *GFPm* (mentioned above) transgenic plants, which were identified using *GFPm*-specific primers at the genome and transcript levels (Supplementary Fig. S5A, B). We then performed a fresh round of GFPm HDR experiments by PEG-mediated co-transformation, using the protoplasts of transgenic tobacco *GFPm*-5, which possess a higher expression level of *GFPm* transcript. The *GFPm*-5 protoplasts transformed with the corresponding plasmids displayed visible fluorescence, while the untransformed protoplasts did not (Supplementary Fig. S5C). Fluorescence results revealed that the HDR emerged in transformed protoplasts. PCR amplification and Sanger sequencing using nuclear DNA, which was extracted from the sorted fluorescence cells by flow cytometry as templates, confirmed the precise boundaries of the HDR events occurring in the nucleus (Supplementary Fig. S5D). Those results showed that the HDR in the CvDT gene editing system occurred in nucleus.

### DNA linker improves HDR efficiency

**Fig. 3.**
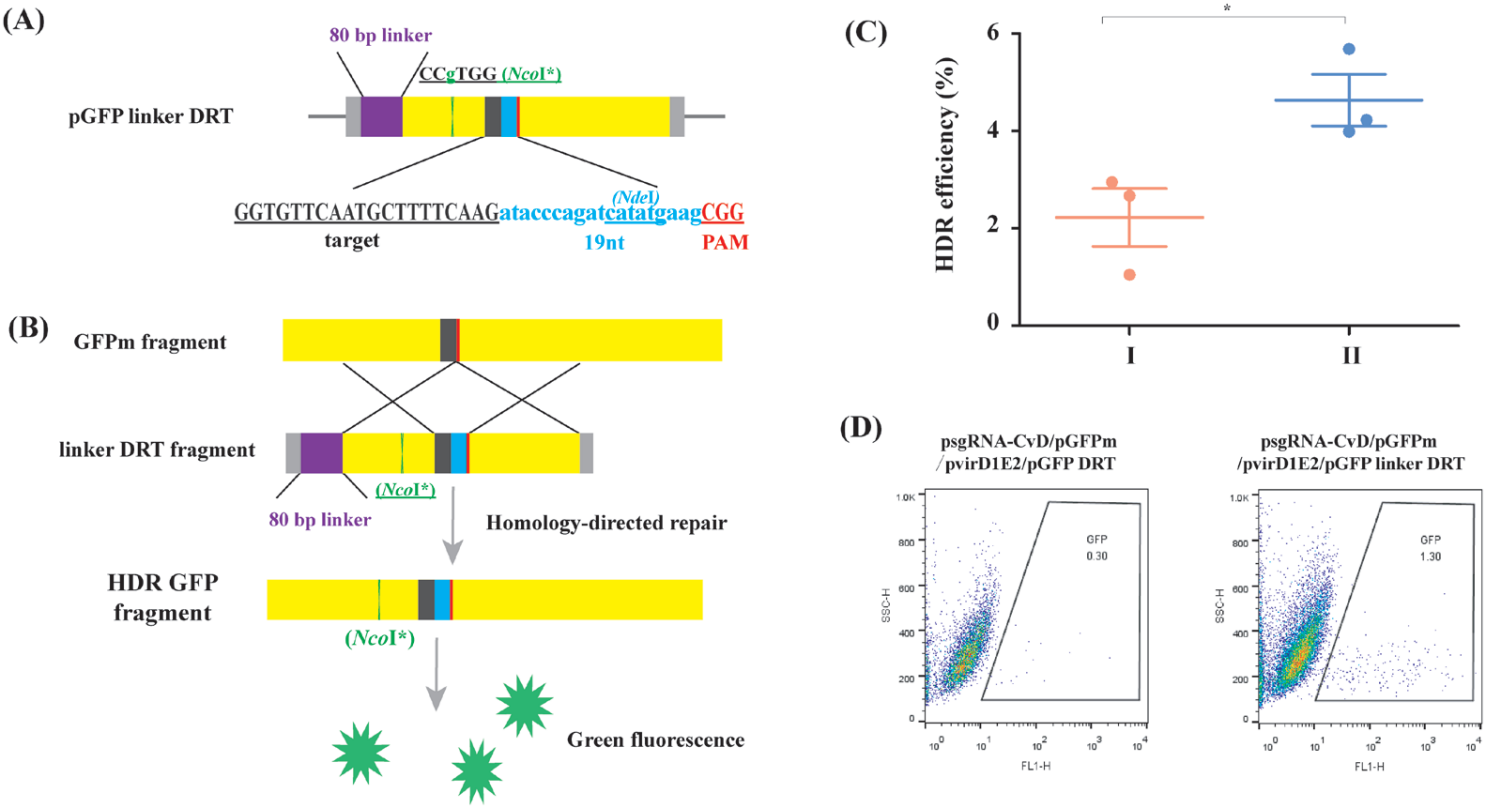
A linker separating the VirD2 recognition sequence and homology region in the repair template increases the efficiency of HDR. (A) Structure of the pGFP linker DRT construct with the 80 bp linker shown in a purple box. The grey bars are the LB and RB sequences. The sgRNA target sequences are shown below the construct in black uppercase letters, the PAM in red uppercase letters, and the 19 nt deletion in blue lowercase letters, containing the *Nde*I site underlined in bule. *Nco*I* represent *Nco*I with a synonymous mutation. (B) Schematic of GFPm and the repair template (GFP linker DRT) to restore GFP activity. The 80 bp linker is shown in a purple box. (C) HDR frequencies in tobacco protoplasts transformed with GFP DRT or GFP linker DRT based on deep amplicon sequencing. I: psgRNA-CvD+pGFPm+pVirD1E2+pGFP DRT; II: psgRNA-CvD+pGFPm+pVirD1E2+pGFP linker DRT (*n*=3; values are means ±SD; asterisks indicate significant differences (Student’s *t*-test, *P<0.05). (D) The abundance of GFP^+^ tobacco protoplasts determined by flow cytometry. The events falling within the GFP sorting gate are framed in a trapezoid box.

The results thus far appeared to support our hypothesis that the direct delivery of the protected donor template to the repair site by VirD1, VirD2, and VirE2 enhances the efficiency of HDR. We speculated that the efficiency could be improved even further by optimizing the positioning of the donor template in the repair complex. We therefore modified the template by inserting an 80 bp linker between the VirD2 recognition sequence and the homology region spanning the DSBs (Fig. 3A; Supplementary Fig. S1C) and compared this to the original template in a fresh round of GFPm HDR experiments (Fig. 3B; Supplementary Table S4). Amplicon sequencing revealed that the inclusion of a linker system CvDTL (CvD, VirD1, VirE2, and a DNA linker) increased the efficiency of HDR by 2.1-fold compared with the DRT CvDT system without a linker (Fig. 3C; Supplementary Table S4), and these results were supported by flow cytometry analysis (Fig. 3D; Supplementary Fig. S6).

### Evaluation of HDR efficiency of the CvDTL editing system in endogenous gene editing in rice

**Fig. 4.**
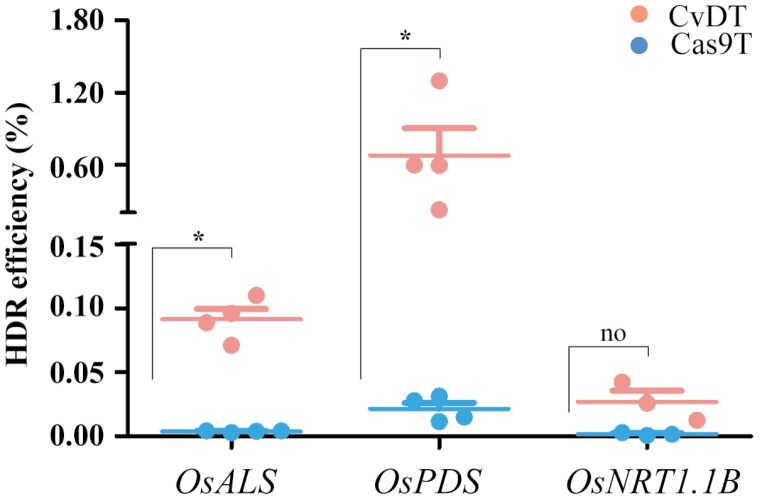
HDR efficiency ratios achieved when targeting three endogenous rice genes based on deep amplicon sequencing. The *OsALS*, *OsPDS* and *OsNRT1.1B* genes were edited using the CvDT and Cas9 systems (values are means ±SD; *n*=4; asterisks indicate significant differences (Student’s *t*-test, *P<0.05); ns: not significant).

Having demonstrated that the enhanced editing system combining CvD, VirD1, VirE2, and a DNA linker can increase the efficiency of HDR on a test sequence in tobacco protoplasts, we next turned our attention to the practical application of the system by targeting endogenous rice genes. Accordingly, we selected the three rice genes *ACETOLACTATE SYNTHASE* (*ALS*), *PHYTOENE DESATURASE* (*PDS*), and *NITROGEN TRANSPORTER 1.1 B* (*NRT1.1B*), and designed suitable sgRNAs targeting specific exons (Supplementary Fig. S7; Supplementary Table S5). The three corresponding donor repair templates were designed to introduce specific point mutations as well as to create/destroy restriction sites for subsequent verification (Supplementary Fig. S8; Supplementary Table S5). We compared the efficiency of the CvDTL and Cas9TL (Cas9, VirD1, VirE2 and a DNA linker) systems by PEG-mediated co-transformation of rice protoplasts. Deep amplicon sequencing revealed HDR frequencies of 9.1 × 0^–4^ (*ALS*), 6.8 × 10^–3^ (*PDS*), and 2.7 × 10^–4^ (*NRT1.1B*) for the CvDTL system, which were an order of magnitude higher than 3.8 × 10^–5^ (*ALS*), 2.1 × 10^–4^ (*PDS*) and 1.7 × 10^–5^ (*NRT1.1B*) achieved using the Cas9TL system, which were shown to be increased 24-, 32- and 16-fold, respectively, in the CvDTL system compared with the corresponding Cas9TL system (Fig. 4; Supplementary Table S5). Results of digital droplet PCR (ddPCR) analysis were consistent with the deep amplicon sequencing. These results confirmed that the HDR efficiency of endogenous gene editing in rice using the CvDTL system was higher than that with the Cas9TL system (Supplementary Fig. S9).

### *OsPDS* HDR precise editing through the CvDTL editing system

**Fig. 5.**
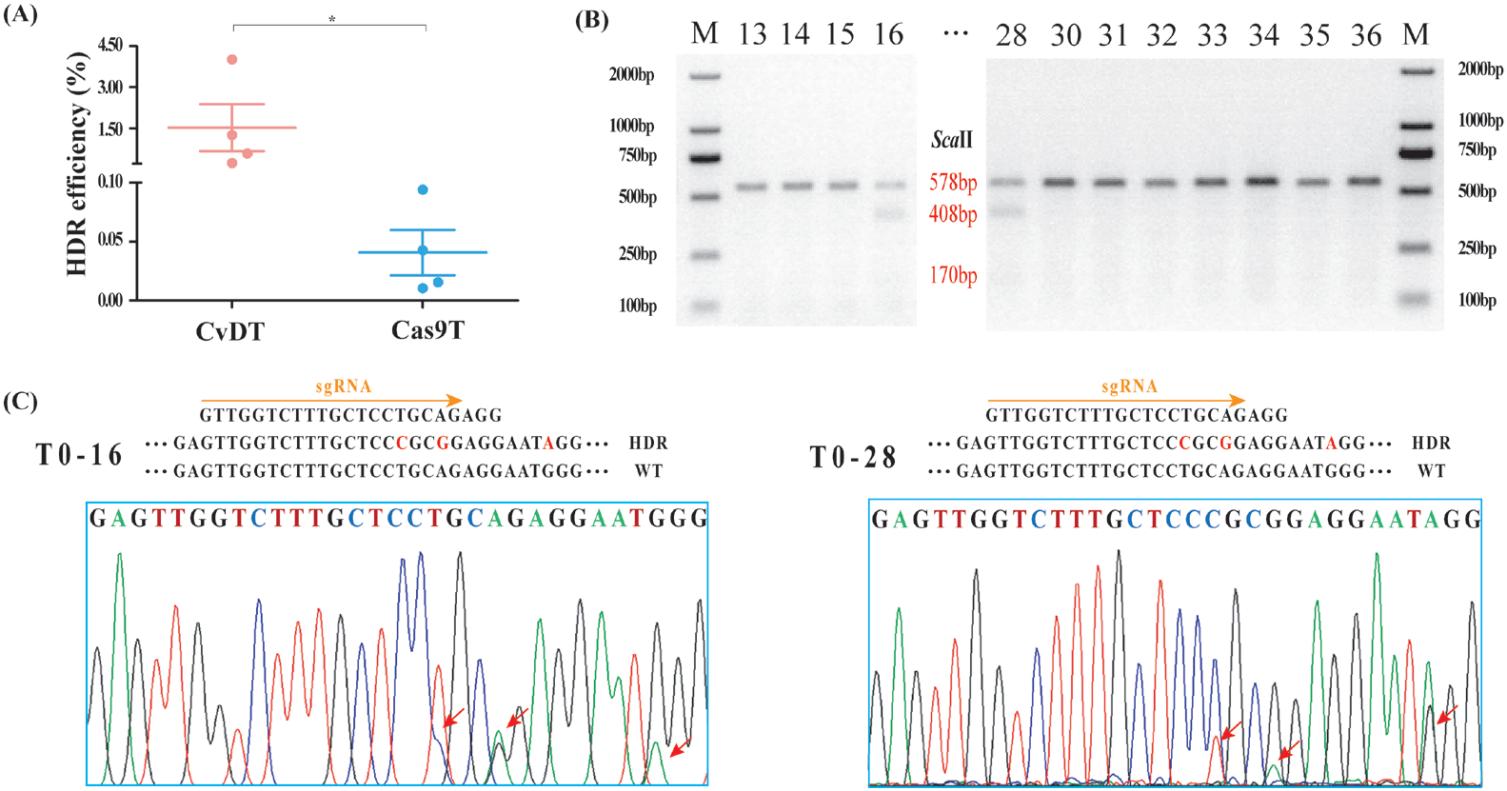
Generation of stable, genome edited rice plants at the *OsPDS* loci. (A) Summary of *OsPDS* HDR ratios in genome-edited rice hygromycin-resistant callus based on deep amplicon sequencing (*n*=4; values are means ±SD; asterisks indicate significant differences (Student’s *t*-test, *P<0.05). (B) PCR restriction enzyme digestion assay (PCR-RE) analysis of *OsPDS* genotypes in regenerated plantlets. The PCR products were digested with S*ca*II, and successful HDR events were revealed by the presence of two bands (lines 16 and 28). M: DL2000 Maker. (C) Sanger sequencing of selected T_0_ transgenic rice plants (*OsPDS* locus). Lines 16 and 28 both appear heterozygous for a precise HDR event.

Given that *PDS* showed higher HDR efficiency in rice protoplasts, we selected this target for genome editing in rice plants. We bombarded embryo-derived callus with the CvDTL system components (or Cas9TL) containing a *HPT* expression cassette for hygromycin selection. After two rounds of selection, we recovered ~200 resistant calli, samples of which were mixed for deep amplicon sequencing. This revealed an HDR efficiency of 1.5 × 10^–2^ for the CvDTL system, compared with 4.1 × 10^–4^ for Cas9TL (Fig. 5A). The superiority of the CvDTL system was confirmed by ddPCR analysis (Supplementary Fig. S10). After 12 weeks in culture, the callus recovered from experiments with both the CvDTL and Cas9TL systems gave rise to many albino plants (Supplementary Fig. S11), confirming the disruption of the *PDS* gene, but not distinguishing between HDR and NHEJ events. 

To detect the HDR events, DNA extracted from 100 regenerated plantlets as a group was used for deep amplicon sequencing. We obtained 32 different amplicons, which added the corresponding barcodes and platform-specific adaptor sequences. Sequencing revealed that HDR events occurred in most barcode groups, and were most frequent in barcode group F6/R4 from the CvDTL system, reaching 9.2 × 10^–3^ (Supplementary Table S6); therefore, we characterized 100 plantlets from this group using a combination of PCR amplification and restriction digests (Fig. 5C). Two plants heterozygous for HDR events were identified based on the partial digestion of amplicons by *Sac*II (Fig. 5B). Both plants with HDR events were confirmed by single plant Sanger sequencing (Fig. 5C). However, we did not obtain homozygous plants with the HDR/HDR genotype from T_1_ seedlings of the two heterozygous plants, possibly due to homozygous lethality of *PDS* HDR. Notably, in sequencing a single plant of the F6/R4 group barcode, we discovered that various *PDS* mutations in these adult regenerating plants contained wild type, heterozygous and chimeric, but not homozygous and biallelic genotypes, due to mutant homozygous lethality. Genotyping analysis results showed that 75.0% of the T_0_ plants were wild type, 22.5% were heterozygous, and 2.5% were chimeric plantlets. Together, these results confirmed that the CvDTL editing system increases efficiency and precise gene editing via HDR in stable transformants.

### *OsALS* HDR precise editing with CvDTL system confers plant tolerance to herbicides

**Fig. 6.**
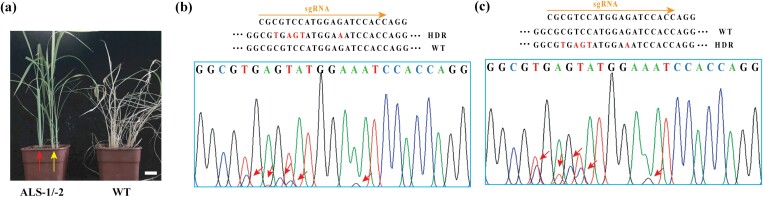
Generation of stable, genome edited rice plants at the *OsALS* loci. (A) Phenotype of T_0_ rice seedlings following the exogenous application of 0.07 mg l^–1^ imazamox. Two-week old genome-edited plants were used for exogenous application of imazamox (ALS-1 and ALS-2 in the same pot, indicated by red and yellow arrows, respectively); the genome-edited plants showed greater herbicide tolerance than wild type (WT) controls at 7 d after exogenous application of imazamox. Scale bar=1 cm. (B, C) Sanger sequencing of selected T_0_ transgenic rice plants (*OsALS* locus). Lines ALS-1 (left) and ALS-2 (right) both appear heterozygous for a precise HDR event.

We also evaluated the *ALS* gene as a target in whole plants because this is a model for the use of HDR to introduce point mutations. A rice *als* mutant with the substitution A96V was previously shown to confer tolerance to imazamox herbicides (Shimatani *et al.*, 2018). We therefore modified the repair template for *ALS* to contain five point mutations, including the A96V mutation and a silent mutation to remove the *Nco*I site (Supplementary Fig. S8). We bombarded embryo-derived calli with the CvDTL system components (or Cas9TL), then regenerated the calli under dual selection for hygromycin and imazamox. Only four resistant callus cultures were recovered from 1560 bombarded explants, two of which regenerated into plantlets that were regarded as two independent transformants, named *ALS*-1 and *ALS*-2 (Fig. 6A). PCR amplicon sequencing showed that the two transformants were heterozygous for the allele replacement, confirming that the CvDTL system had induced HDR at the *ALS* locus (Fig. 6B). The exogenous application of 0.07 mg l^–1^ imazamox did not affect the growth of the mutants, whereas wild-type control plants were rapidly killed, confirming that the replacement *als* allele was functional in *planta* (Fig. 6A). Subsequently, a homozygous *ALS* mutant with HDR/HDR genotype was obtained through screening using imazamox and sequencing from T_1_ seedlings of two heterozygous regenerating plants, confirming that the CvDTL system is an efficient and precise gene editing tool to produce stable mutants in plants via the HDR pathway.

Finally, the specificity of the CvDTL system was evaluated using CRISPR-GE (CRISPR-Genome Editing, http://skl.scau.edu.cn/; Xie *et al.*, 2017) to map the potential off-targets of the *OsPDS* and *OsALS* genes. The potential off-target sites in the ‘Nipponbare’ genome of sgRNAs targeting *OsPDS* and *OsALS* genes were predicted, as shown in Supplementary Table S7. The off-target sequence sites were individually examined by amplicon sequencing. We found no evidence of mutations at any of the potential off-target sites in the HDR plants (Supplementary Table S7). These results show that the CvDTL system has minimal potential for off-target activity, as previously reported for Cas9 alone (Shan *et al.*, 2013).

In this study, we have developed a hybrid CRISPR/Cas9 and T-DNA delivery system using the components of the latter to increase the efficiency of precise genome editing via HDR. When we replaced Cas9T with the new CvDT system, we achieved a striking 52-fold increase in the efficiency of HDR when testing a GFP construct, and up to a 32-fold increase when targeting the endogenous *OsPDS* gene. We were able to optimize the system by introducing a linker to increase precise editing efficiency. Further optimization may be possible by modifying the linker length, the relative amounts of the protein components and repair template, and other parameters.

## Discussion

The CRISPR/Cas9 editing system has been widely used to produce gene mutations, *cis*-element mutations, gene expression repression or activation, and genome structure mutation (Oliva *et al.*, 2019;Beying *et al.*, 2020;Gao, 2021;Hendelman *et al.*, 2021; Liu *et al.*, 2021). However, it is difficult to achieve precise genome editing using tailored DNA donors via the HDR pathway, even if some researchers have optimized and improved this editing system to increase efficiency of precise genome editing. Thus, novel precise genome editing systems using the CRISPR/Cas9 editor via the HDR pathway remain explored. Here, we developed a CvDTL genome editing system to increase the efficiency of precise genome editing via the HDR pathway by using the synergy between CRISPR/Cas9 and *Agrobacterium* virulence proteins.

In the present study, we combined Cas9 and VirD2 to form CvD fusing protein, which conferred its ability of making DSBs and tethering DNA donor (Fig. 1A; Supplementary Table S3). Combination of CvD, VirD1, and VirE2, as well as sgRNA and optimized-DNA donor (CvDTL editing system) showed remarkable increasing efficiency of precise genome editing via the HDR pathway, compared with a Cas9 combination, verifying that the CvD editor is efficient at precise genome editing via the HDR pathway (Fig. 3C, D; Supplementary Table S4). Recently, Ali *et al.* (2020) demonstrated that the ability of CvD to make DSBs is comparable with Cas9, and carries a DNA donor to increase the ratio of HDR events compared with Cas9. This is repeated in the present study, in that CvD fused protein is indeed efficient in precise genome editing via the HDR pathway (Supplementary Fig S3). However, we found that VirD1 played an important role in increasing HDR events (Figs 1E, 2E; Supplementary Table S3), due to VirD1 helping VirD2 cleavage to a specific recognized site in this study, whereas the recent literature did not provide VirD1 function. Thus, relative ratios of HDR events in our CvDT system compared with Cas9T control is higher by 52-fold, partially due to the help of VirD1, whereas in a recent report, the fusion of Cas9-VirD2 only increased the ratios of HDR events 5- to 6-fold, compared with Cas9 (Ali *et al.*, 2020). These results demonstrated that CvD fused protein with the help of VirD1 can form CvD-DNA complex and carry the DNA donor for highly effective precise genome editing via the HDR pathway.

We found that VirE2 can significantly increase HDR events in the CvDT editing system. VirE2 can protect T-DNA from nuclease digestion. Thus, in the CvDT editing system, VirE2 possibly binds to DRT to avoid digestion, resulting in significant increase of precise genome editing efficiency via the HDR pathway (Figs 1E, 2E; Supplementary Table S3). Several reports showed that various chemically modified DRTs have been developed to enhance HDR efficiencies compared with donors without modification (Gutierrez-Triana *et al.*, 2018;Ali *et al.*, 2020;Lu *et al.*, 2020). For example, 5’ and 3’ phosphorothioate end-modified repair templates was able to enhance the 4-fold HDR efficiency, compared with the unmodified repair templates, by stabilizing repair templates in the plant nucleus (Ali *et al.*, 2020). The RNA::TEG (15 nucleotide 2’ OMe-RNA fused to triethylene glycol)-modified dsDNA donors increased HDR efficiency by 2- to 5-fold in mammalian cells, compared with unmodified donors (Ghanta *et al.*, 2021). Harmsen *et al.* (2018) reported that the single-stranded oligonucleotides (ssODNs) 3’ end with phosphorothioate linkages was able to protect the 3’ end of the ssODNs from degradation by exonucleases, leading to the conclusion that the donor template ssODN is available in proximal DSBs. However, the protected DRTs can be also digested by endonucleases due to their naked internal sequences, unlike VirE2 coated sequences. Therefore, native VirE2-protected DRTs could be more stable in cells compared with other end-modified protection.

In the present study, the CvDT editing system showed a lower ratio of HDR events compared with other reports (Gutschner *et al.*, 2016;Aird *et al.*, 2018; Gutierrez-Triana *et al.*, 2018). However, the relative ratio of HDR events in the CvDT editing system to Cas9T in our study was higher than the reports mentioned above. For exogenous *GFPm* gene editing, the HDR efficiency of the CvDT editing system is higher by up to 52-fold compared with that in Cas9T (Fig. 2E; Supplementary Table S3). Recent literature documented that the Cas9-VirD2 system without the help of other Vir proteins enhances the ratio of HDR editing by up to 5- to 6-fold compared with Cas9 (Ali *et al.*, 2020). Furthermore, Cas9-PCV fusion protein can increase HDR efficiency by nearly 30-fold compared with that of Cas9 protein, indicating that PCV is a better protein covalently bound to the DRT (Aird *et al.*, 2018). Chu *et al.* (2015) reported that an inhibitor co-expressed with the Cas9 system increased the efficiency of HDR by 4- to 5-fold through suppression of the KU70 and DNA ligase IV activity. In this study, the HDR efficiency in the CvDT/CvDTL editing system was repeated at least three times (Fig. 3C, D; Supplementary Table S4). Thus, the lower ratios of precise genome editing via the HDR pathway in the CvDTL editing system, compared with other reports is possibly due to different manipulations regarding plant genetic transformation in various laboratories, which result in different efficiencies to obtain GFP^+^ positive cell, calli, or regenerating plantlets.

Notably, we were able to further optimize the CvDTL editing system to increase precise editing efficiency, including the linker length modification, the relative amounts of the protein components and repair template, etc. More importantly, the *A. tumefaciens* T-DNA transfer system consists of 24 virulence proteins, among which VirD1, VirD2, VirD5, VirE2, VirE3, and VirF are known to enter into host cells (Rogowsky *et al.*, 1990;Sakalis *et al.*, 2014). We tested only three of these proteins and increased the efficiency of HDR by an order of magnitude, but additional Vir components may increase the system’s efficiency even further, providing greater opportunities for the genome editing of plants in the future.

The CvDTL editing system was evaluated and developed through PEG-mediated co-transformation of tobacco protoplasts and bombarded rice embryos-derived calli. We found that the higher efficiency of precise gene editing via the HDR pathway was obtained by using the CvDTL editing system, compared with the Cas9TL editing system, although three rice targeting genes showed different increases in HDR event ratios (Fig. 4; Supplementary Table S5). The CvDTL editing system components, CvD, VirD1, VirE2, sgRNA, and the DRT with linker, can be tailored as a tool kit, and easily employed to conduct precise genome editing via the HDR pathway, mainly through biolistic bombardment-mediated transformation. If the CvDTL editing system was to be improved and optimized for *Agrobacterium*-mediated genetic transformation, it would be widely used in precise genome editing in plants. The CvDTL editing system may then be employed to conduct gene editing in yeast, fungi and animals.

## Supplementary data

The following supplementary data are available at *JXB* online. 

Fig. S1. Sequences of *GFPm* and the DNA repair template.

Fig. S2. PEG-mediated co-transformation of tobacco protoplasts analysed by flow cytometry.

Fig. S3. PEG-mediated co-transformation with psgRNA-CvD/pGFPm/pVirD1E2/pDRT and psgRNA-CvD/pGFPm/pDRT.

Fig. S4. Visual representation of the multi-cycle nested PCR.

Fig. S5. Identification *GFPm* transgenic plants HDR events by PCR, microscopy and Sanger sequencing.

Fig. S6. Analysis of tobacco treated and untreated protoplasts for flow cytometry and fluorescence.

Fig. S7. Structure of the psgRNA-CvD and psgRNA-Cas9 vectors used for rice genome editing.

Fig. S8. Structure of the pDRT vectors and sequences.

Fig. S9. Detection of *OsPDS* wild type and HDR genotypes in transformed protoplasts by ddPCR.

Fig. S10. Detection of *OsPDS* wild type and HDR genotypes in embryo-derived rice callus by ddPCR.

Fig. S11. The albino phenotype of *OsPDS-*edited plants.

Dataset S1. Complete sequences of the gRNA expression vectors pAtU6-gRNA for tobacco and pOsU6c-gRNA for rice.

Dataset S2. Complete sequences of the plant codon-optimized *Cas9* and *CvD* expression vectors.

Dataset S3. Complete sequences of the plant codon-optimized *VirD1* and *VirE2* expression vectors.

Table S1. The primers used in this study.

Table S2. Targets and sgRNA sequences in the *GFPm* gene.

Table S3. The HDR ratio of the *GFP* gene following transformation with various combinations of vectors.

Table S4. Transformation with the full CvD components and DRTs with and without linkers, and the corresponding HDR ratios.

Table S5. Endogenous rice target genes and the corresponding sgRNA sequences, diagnostic restriction sites and HDR editing ratios.

Table S6. *OsPDS* gene HDR ratio in 32 groups of barcodes.

Table S7. Potential off-target sites of the sgRNAs used in this study.

erad096_suppl_Supplementary_Figures_S1-S11_Datasets_S1-S3_Tables_S1-S7

## Data Availability

All relevant data can be found within the article and its supplementary data published online.
