# Supplementary material for: CRISPR/Cas9 and Agrobacterium tumefaciens virulence proteins synergistically increase efficiency of precise genome editing via homology directed repair in plants
Source: J Exp Bot. 2023 Mar 15;74(12):3518–30. doi: 10.1093/jxb/erad096 (PMC10797490; doi:10.1093/jxb/erad096)
Supplement: erad096_suppl_Supplementary_Figures_S1-S11_Datasets_S1-S3_Tables_S1-S7 [file erad096_suppl_supplementary_figures_s1-s11_datasets_s1-s3_tables_s1-s7.pdf]

SUPPLEMENTAL INFORMATION

CRISPR/Cas9 and *Agrobacterium tumefaciens* virulence proteins synergistically increase efficiency of precise genome editing via homology directed repair in plants

Ye Tang, Zhennan Zhang, ZhiyuanYang, Jiahe Wu*

State Key Laboratory of Plant Genomics, Institute of Microbiology, Chinese Academy of Sciences, Beijing, China

* Correspondence should be addressed to Jiahe Wu (wujiahe@im.ac.cn)

(A)

GFPm (406bp)

```
GAACTTTTCACTGGAGTTGTCCCAATTCTTGTTGAATTAGATGGTGATGTTAATGGGCACA
AATTTTCTGTCAGTGGAGAGGGTGAAGGTGATGCAACATACGGAAAACTTACCCTTAAAT
TTATTTGCACTACTGGAAAACTACCTGTTCCATGGCCAACACTTGTCACTACTTTCTGTTAT
                             NcoI
GGTGTTCAATGCTTTTCAAG-------------------CGGCACGACTTCTTCAAGAGCGC
CATGCCTGAGGGATACGTGCAGGAGAGGACCATCTTCTTCAAGGACGACGGGAACTACAA
GACACGTGCTGAAGTCAAGTTTGAGGGAGACACCCTCGTCAACAGGATCGAGCTTAAGGG
AATCGATTTCAAGGAGGACGGAAACATCCTCGGCCACAAGTTGGAATACAACTACAACTC
```

(B)

GFP DRT (475bp)

```
GTTTACCCGCCAATATATCCTGTCAGAACTTTTCACTGGAGTTGTCCCAATTCTTGTTGAATTAG
ATGGTGATGTTAATGGGCACAAATTTTCTGTCAGTGGAGAGGGTGAAGGTGATGCAACATACG
                                                 NcoI*
GAAAACTTACCCTTAAATTTATTTGCACTACTGGAAAACTACCTGTTCCgTGGCCAACACTTGTC
                                         NdeI
ACTACTTTCTGTTATGGTGTTCAATGCTTTTCAAGatacccagatcatatgaagCGGCACGACTTCTTC
AAGAGCGCCATGCCTGAGGGATACGTGCAGGAGAGGACCATCTTCTTCAAGGACGACGGGAAC
TACAAGACACGTGCTGAAGTCAAGTTTGAGGGAGACACCCTCGTCAACAGGATCGAGCTTAAG
GGAATCGATTTCAAGGAGGACGGAAACATCCTCGGCCACAAGTTGGAATACAACTACAACTCT
GGCAGGATATATTGTGGTGTAAAC
```

(C)

GFP linker DRT (555bp)

```
GTTTACCCGCCAATATATCCTGTCAATGTTACGTCCTGTAGAAACCCCAACCCGTGAAATCAAAA
AACTCGACGGCCTGTGGGCATTCAGTCTGGATCGCGAAAAGAACTTTTCACTGGAGTTGTCCCA
ATTCTTGTTGAATTAGATGGTGATGTTAATGGGCACAAATTTTCTGTCAGTGGAGAGGGTGAAG
                                                         NcoI*
GTGATGCAACATACGGAAAACTTACCCTTAAATTTATTTGCACTACTGGAAAACTACCTGTTCCg
                                                   NdeI
TGGCCAACACTTGTCACTACTTTCTGTTATGGTGTTCAATGCTTTTCAAGatacccagatcatatgaag
CGGCACGACTTCTTCAAGAGCGCCATGCCTGAGGGATACGTGCAGGAGAGGACCATCTTCTTC
AAGGACGACGGGAACTACAAGACACGTGCTGAAGTCAAGTTTGAGGGAGACACCCTCGTCAA
CAGGATCGAGCTTAAGGGAATCGATTTCAAGGAGGACGGAAACATCCTCGGCCACAAGTTGGA
ATACAACTACAACTCTGGCAGGATATATTGTGGTGTAAAC
```

Fig. S1 Sequences of GFPm and the DNA repair template.

(A) The partial sequence of GFPm (406 bp) shows the 19-bp deletion as orange dashes and the N*co*I site underlined. **(B)** The complete sequence of the GFP DNA repair template (DRT) shows the RB and LB in gray, the 19-bp repair in orange lower case (including an underlined N*de*I site), and the synonymous mutation destroying the (underlined) N*co*I site in green lower case are maked as N*co*I*. **(C)** The complete sequence of the GFP linker DRT is labeled in the same manner as **(B)** but with the additional 80-bp shown in blue. In all three sequences, the sgRNA target is framed in a red box and the PAM is shown in red.

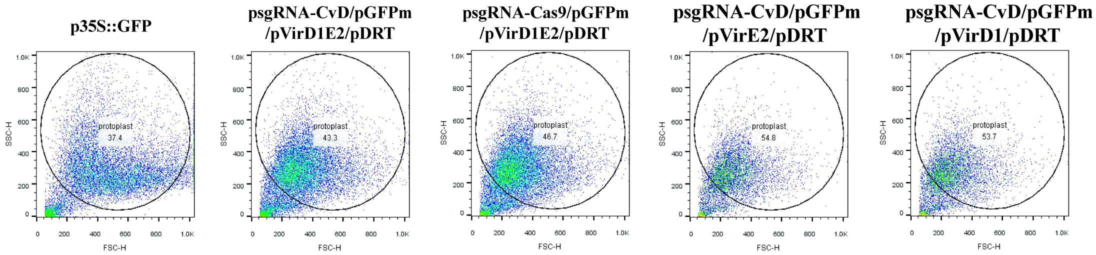

Fig. S2 PEG-mediated co-transformation of tobacco protoplasts was analysed by flow cytometry.

The PEG-mediated co-transformation of tobacco protoplasts with p35S::GFP, psgRNA-CvD/pGFPm/pVirD1E2/pDRT, psgRNA-Cas9/pGFPm/pVirD1E2/pDRT, psgRNA-CvD/pGFPm/pVirE2/pDRT and psgRNA-CvD/pGFPm/pVirD1/pDRT was analyzed by flow cytometry. Approximately 10,000 events are presented in each plot. The events falling within the protoplast sample sorting gate are framed in a black circle.

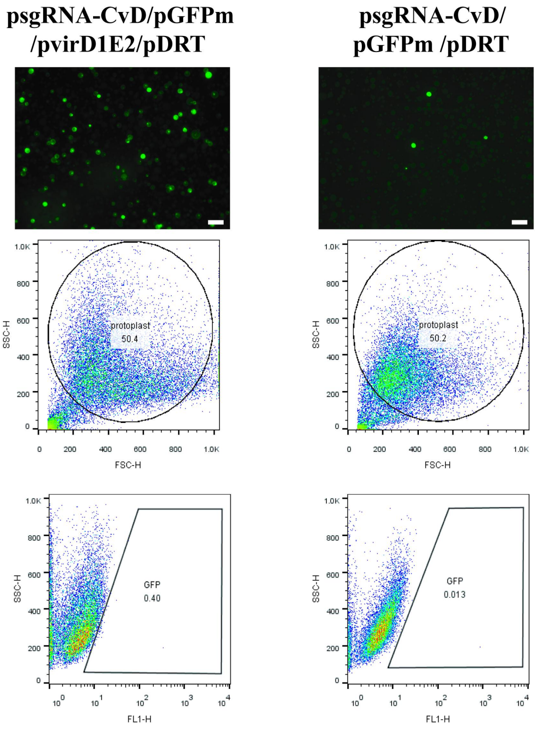

Fig. S3 PEG-mediated co-transformation with psgRNA-CvD/pGFPm/pVirD1E2/pDRT and psgRNA-CvD/pGFPm/pDRT.

The abundance of GFP^{+} tobacco protoplasts as determined by microscop (upper row, scale bar = 100 µm) and flow cytometry (lower row).

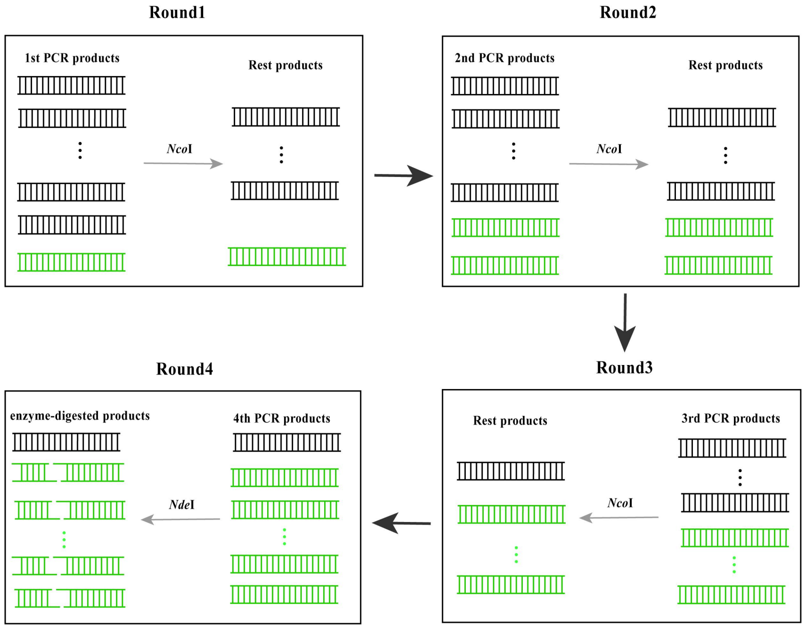

Fig. S4 Visual representation of the multi-cycle nested PCR.

The black and green double helices represent the original and HDR-repaired sequences, respectively.

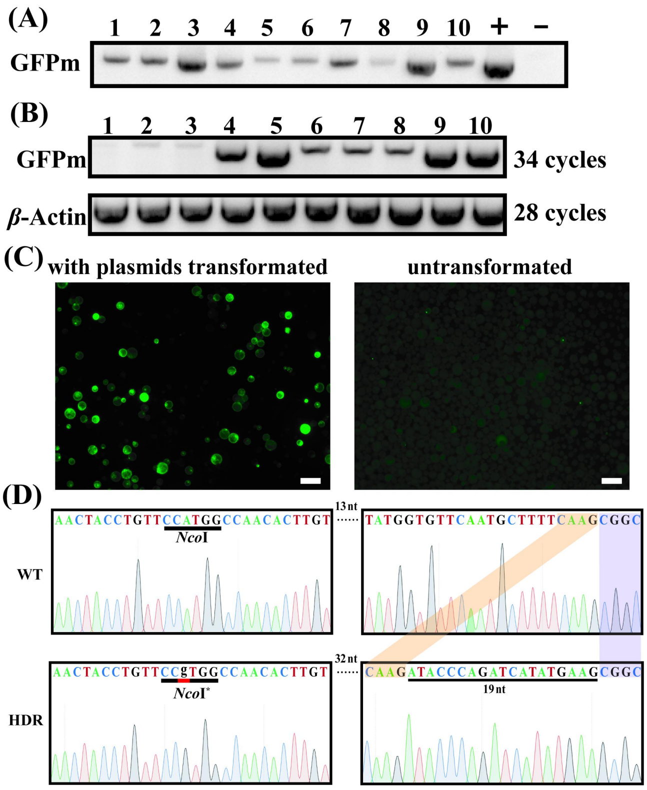

Fig. S5 Identification *GFPm* transgenic plants HDR events by PCR, microscopy and Sanger sequencing.

(A) PCR identified the *GFPm* transgenic plants in genomic level. Lanes 1-10, each different *GFPm* plant, +, plasmid control. -, negative control. **(B)** RT-PCR identified the *GFPm* transgenic plants in transcript level. *β*-Actin used as a reference gene. **(C)** The abundance of GFP^+ tobacco protoplasts as determined by microscopy (scale bar = 100 μm). **(D)** Sanger sequencing chromatogram analysis the HDR events. The N*co*I* represent N*co*I with a synonymous mutation.

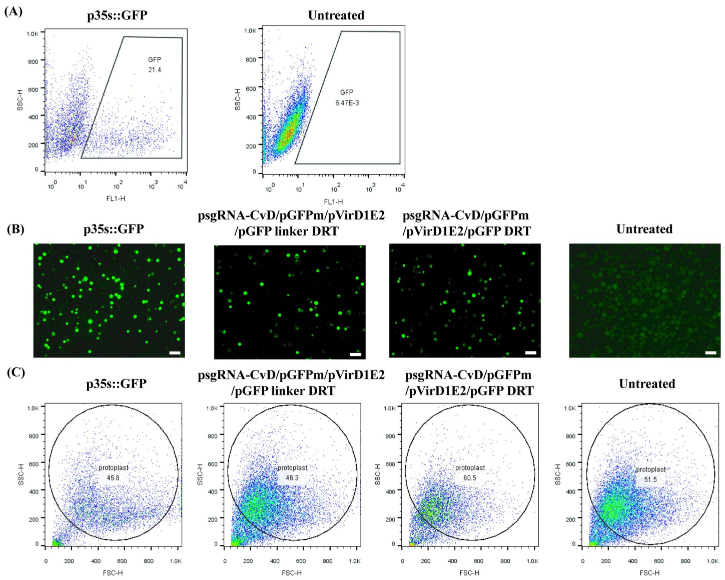

Fig. S6 Analysis of tobacco treated and untreated protoplasts of the flow cytometry and fluorescence.

(A) The abundance of GFP^{+} tobacco protoplasts determined by flow cytometry for p35S::GFP and untreated. **(B)** Microscopy reveals the fluorescence of the protoplasts transformed with p35S::GFP, psgRNA-CvD/pGFPm/pVirD1E2/pGFP linker DRT, or psgRNA-CvD/pGFPm/pVirD1E2/pGFP DRT. Scale bars = 100 μm. **(C)** Flow cytometry reveals the events falling within the protoplast sample sorting gate (within the black circle).

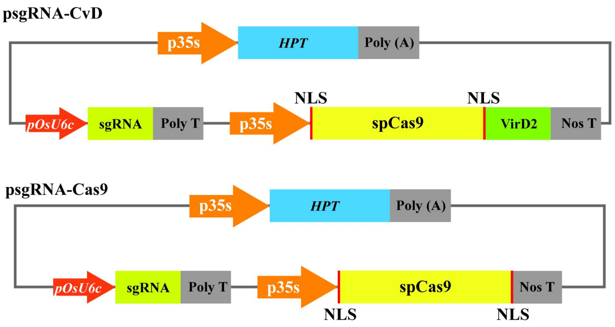

Fig. S7 Structure of the psgRNA-CvD and psgRNA-Cas9 vectors used for rice genome editing.

The *CvD* and *Cas9* genes were expressed under the control of CaMV35S promoter and the NOS terminator, the sgRNA was driven by the OsU6c promoter and poly-T terminator, the *hpt* genes were expressed under the control of CaMV35S promoter and the CaMV Poly (A) terminator.

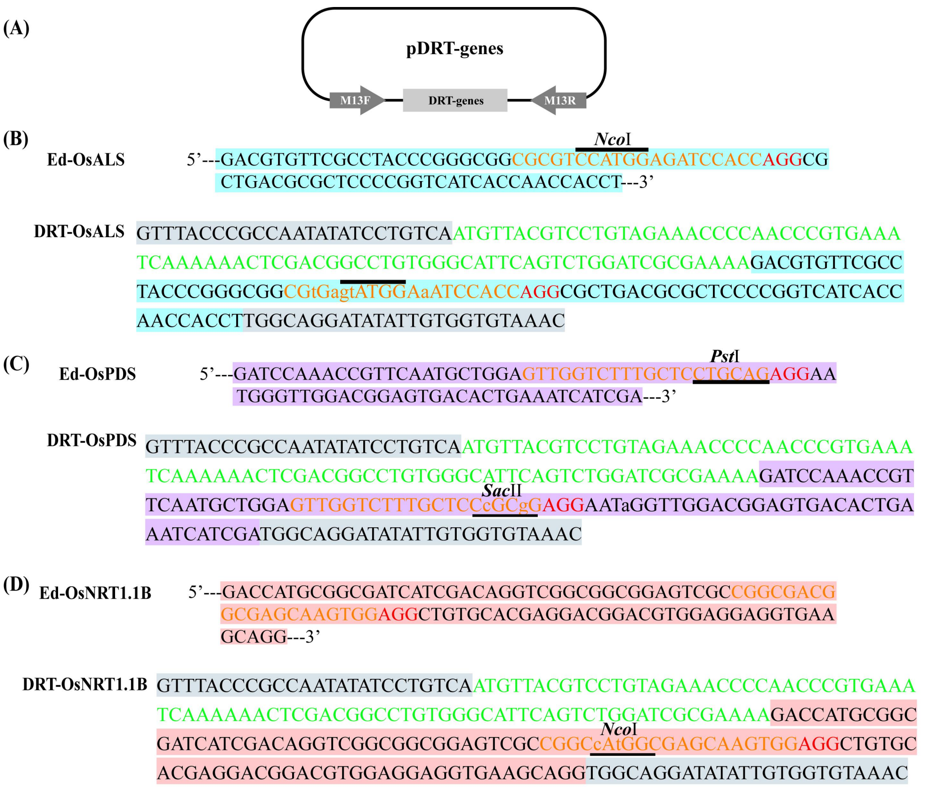

Fig. S8 Structure of the pDRT vectors and sequences.

(A) The DRT sequence of different genes were inserted into vector pClone007 to generate vectors pDRT-genes. **(B)** The edited *OsALS* gene (Ed-OsALS) and DRT-OsALS sequence. The sgRNA target sequence is shown in orange with an underlined N*co*I site. The complete OsALS DRT sequence includes five mutations shown in lower case, including the A96V mutation and the abolition of the N*co*I site. **(C)** The edited *OsPDS* gene (Ed-OsPDS) and DRT-OsPDS sequence. The sgRNA target sequence is shown in orange with an underlined P*st*I site. The complete OsPDS DRT sequence includes three mutations shown in lower case, resulting in premature termination, the elimination of the P*st*I site, and the creation of a S*ac*II site. **(D)** The edited *OsNRT1.1B* gene (Ed-OsNRT1.1B) and DRT-OsNRT1.1B sequence. The sgRNA target sequence is shown in orange. Complete sequences of the OsNRT1.1 DRT includes two nucleoside mutations shown in black lower character in target sequence introducing N*co*I site. PAM are all in red characters.

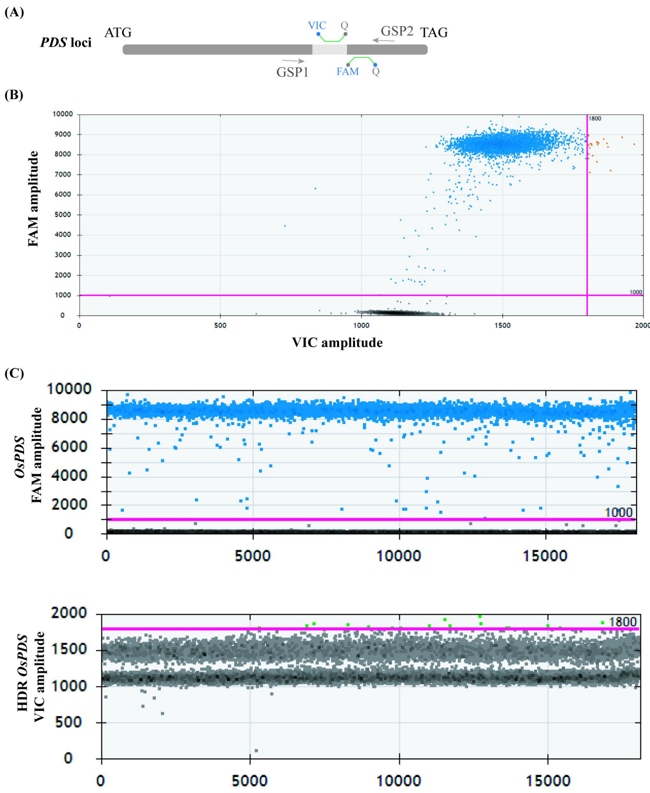

Fig. S9 Detection of *OsPDS* wild-type and HDR genotypes in transformed protoplasts by ddPCR.

(A) Schematic description of the ddPCR process. Primers GSP1 and GSP2 are designed to amplify the *PDS* sequence. The probes are labeled with 5′-VIC and 5′-FAM and are used to detect PCR products representing the mutated *PDS* gene. Q = quencher. **(B)** Two-dimensional fluorescence plots of droplets (amplitude indicated on y-axis) containing *OsPDS* amplicons (FAM labeled; positive droplets are in blue) or *OsPDS* HDR amplicons (VIC labeled; positive droplets are in orange) in rice protoplasts. Negative droplets are shown in black. **(C)** One-dimensional fluorescence plot of droplets (amplitude indicated on y-axis) containing the *OsPDS* gene (FAM labeled; positive droplets are in blue) or *OsPDS* HDR gene (VIC labeled; positive droplets are in green). Negative droplets are shown in black.

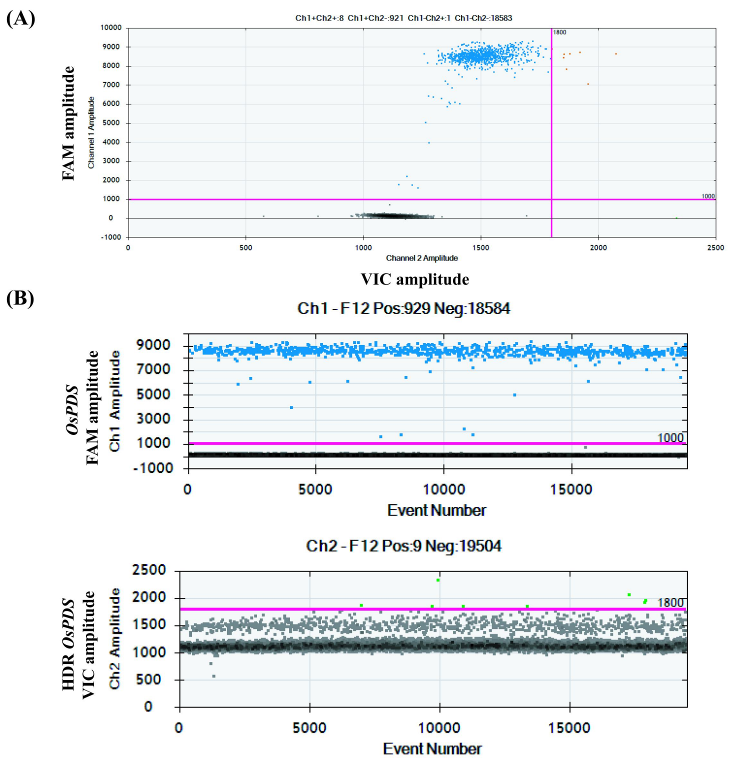

Fig. S10 Detection of *OsPDS* wild-type and HDR genotypes in seed-derived rice callus by ddPCR.

(A) Two-dimensional fluorescence plots of droplets (amplitude indicated on y-axis) containing *OsPDS* amplicons (FAM labeled; positive droplets are in blue) or *OsPDS* HDR amplicons (VIC labeled; positive droplets are in orange) in rice callus. **(B)** One-dimensional fluorescence plot of droplets (amplitude indicated on y-axis) containing the *OsPDS* gene (FAM labeled; positive droplets are in blue) or *OsPDS* HDR gene (VIC labeled; positive droplets are in green). Negative droplets are shown in black.

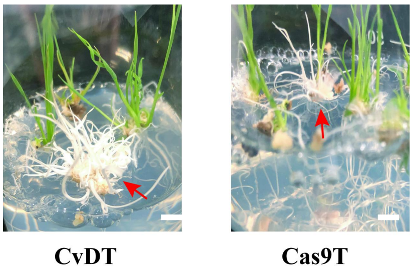

Fig. S11 The *OsPDS*-edited plant albino phenotype.

Phenotype of T0 rice seedlings of *OsPDS* gene editing in CvDT (left) and Cas9T (right) system, scale bar = 1 cm.

(A)

5′-agaaatctcaaaattccggcagaacaattttgaatctcgatccgtagaaacgagacggtcattgttttagttccaccacgattatatttgaaatt
tacgtgagtgtgagtgagacttgcataagaaaataaaatctttagttgggaaaaaattcaataatataaatgggcttgagaaggaagcgaggga
taggcctttttctaaaataggcccatttaagctattaacaatcttcaaaagtaccacagcgcttaggtaaagaaagcagctgagtttatatatggtta
gagacgaagtagtgattgGTGTTCAATGCTTTTCAAGgttttagagctagaaatagcaagttaaaataaggctagtccgttatc
aacttgaaaaagtggcaccgagtcggtgctttttt-3′

Guide sequence insert 5′-attgNNNNNNNNNNNNNNNNNNN-3′
3′-NNNNNNNNNNNNNNNNNNNcaaa-5′

(B)

5′-ctcattagcggtatgcatgttggtagaagtcggagatgtaaataattttcattatataaaaaaggtacttcgagaaaaataaatgcatacgaatt
aattctttttatgtttttaaaccaagtatatagaatttattgatggttaaaatttcaaaaatatgacgagagaaaggttaaacgtacggcatatacttct
gaacagagagggaatatggggtttttgttgctcccaacaattcttaagcacgtaaaggaaaaaagcacattatccacattgtacttccagagata
tgtacagcattacgtaggtacgttttctttttcttcccggagagatgatacaataatcatgtaaacccagaatttaaaaaatattctttactataaaaat
tttaattagggaacgtattatttttacatgacaccttttgagaaagagggacttgtaatatgggacaaatgaacaatttctaagaaatgggcatatg
actctcagtacaatggaccaaattccctccagtcggcccagcaatacaaagggaaagaaatgagggggcccacaggccacggcccactttt
ctccgtggtggggagatccagctagaggtccggcccacaagtggccccttgccccgtgggacggtgggattgcagagcgcgtgggcggaa
acaacagtttagtaccacctcgctcacgcaacgacgcgaccacttgcttataagctgctgcgctgaggctcagGGGTATGGTGGT
GCAATGGGgttttagagctagaaatagcaagttaaaataaggctagtccgttatcaacttgaaaaagtggcaccgagtcggtgctttttt-
3′

Guide sequence insert 5′-tcagNNNNNNNNNNNNNNNNNNN-3′
3′-NNNNNNNNNNNNNNNNNNNcaaa-5′

Supplemental Dataset 1 Complete sequences of the gRNA expression vectors pAtU6-gRNA for tobacco and pOsU6c-gRNA for rice.

(A) The gRNA scaffold sequence (blue) under the control of *Arabidopsis thaliana* U6-1 promoter and poly-T terminator (black lower case after the blue case). The guide sequence is shown in upper case. In the target region, any G acts as a transcriptional start site for the U6-1 promoter, thus all genomic sites of the form 5′-G-N(20)-GG-3′ are targeted. **(B)** The gRNA scaffold sequence (blue) under the control of rice U6c promoter and poly-T terminator (black lower case after the blue case). The guide sequence is shown in upper case. In the target region, any G acts as a transcriptional start site for the U6c promoter, thus all genomic sites of the form 5′-G-N(20)-GG-3′ are preferentially targeted.

(A)

5′-TGAGACTTTTCAACAAAGGGTAATATCCGGAAACCTCCTCGGATTCCATTGCCCAGC
TATCTGTCACTTTATTGTGAAGATAGTGGAAAAGGAAGGTGGCTCCTACAAATGCCATC
ATTGCGATAAAGGAAAGGCCATCGTTGAAGATGCCTCTGCCGACAGTGGTCCCAAAGA
TGGACCCCCACCCACGAGGAGCATCGTGGAAAAAGAAGACGTTCCAACCACGTCTTC
AAAGCAAGTGGATTGATGTGATATCTCCACTGACGTAAGGGATGACGCACAATCCCACT
ATCCTTCGCAAGACCCTTCCTCTATATAAGGAAGTTCATTTCATTTGGAGAGAACACGG
GGGACTCTAGATTAATTAAGcctaagaagaagcggaaggttGGTGTGCCTGCGATGGACAAGAAGT
ACTCCATCGGCCTCGACATCGGCACCAACAGCGTCGGCTGGGCGGTGATCACCGACGA
GTACAAGGTCCCGTCCAAGAAGTTCAAGGTCCTGGGCAACACCGACCGCCACTCCATC
AAGAAGAACCTCATCGGCGCCCTCCTCTTCGACTCCGGCGAGACGGCGGAGGCGACC
CGCCTCAAGCGCACCGCCCGCCGCCGCTACACCCGCCGCAAGAACCGCATCTGCTACC
TCCAGGAGATCTTCTCCAACGAGATGGCGAAGGTCGACGACTCCTTCTTCCACCGCCT
CGAGGAGTCCTTCCTCGTGGAGGAGGACAAGAAGCACGAGCGCCACCCCATCTTCGG
CAACATCGTCGACGAGGTCGCCTACCACGAGAAGTACCCCACTATCTACCACCTTCGTA
AGAAGCTTGTTGACTCTACTGATAAGGCTGATCTTCGTCTCATCTACCTTGCTCTCGCTC
ACATGATCAAGTTCCGTGGTCACTTCCTTATCGAGGGTGACCTTAACCCTGATAACTCC
GACGTGGACAAGCTCTTCATCCAGCTCGTCCAGACCTACAACCAGCTCTTCGAGGAGA
ACCCTATCAACGCTTCCGGTGTCGACGCTAAGGCGATCCTTTCCGCTAGGCTCTCCAAG
TCCAGGCGTCTCGAGAACCTCATCGCCCAGCTCCCTGGTGAGAAGAAGAACGGTCTTT
TCGGTAACCTCATCGCTCTCTCCCTCGGTCTGACCCCTAACTTCAAGTCCAACTTCGAC
CTCGCTGAGGACGCTAAGCTTCAGCTCTCCAAGGATACCTACGACGATGATCTCGACA
ACCTCCTCGCTCAGATTGGAGATCAGTACGCTGATCTCTTCCTTGCTGCTAAGAACCTC
TCCGATGCTATCCTCCTTTCGGATATCCTTAGGGTTAACACTGAGATCACTAAGGCTCCT
CTTTCTGCTTCCATGATCAAGCGCTACGACGAGCACCACCAGGACCTCACCCTCCTCA
AGGCTCTTGTTCGTCAGCAGCTCCCCGAGAAGTACAAGGAGATCTTCTTCGACCAGTC
CAAGAACGGCTACGCCGGTTACATTGACGGTGGAGCTAGCCAGGAGGAGTTCTACAA
GTTCATCAAGCCAATCCTTGAGAAGATGGATGGTACTGAGGAGCTTCTCGTTAAGCTTA
ACCGTGAGGACCTCCTTAGGAAGCAGAGGACTTTCGATAACGGCTCTATCCCTCACCA
GATCCACCTTGGTGAGCTTCACGCCATCCTTCGTAGGCAGGAGGACTTCTACCCTTTCC
TCAAGGACAACCGTGAGAAGATCGAGAAGATCCTTACTTTCCGTATTCCTTACTACGTT
GGTCCTCTTGCTCGTGGTAACTCCCGTTTCGCTTGGATGACTAGGAAGTCCGAGGAGA
CTATCACCCCTTGGAACTTCGAGGAGGTTGTTGACAAGGGTGCTTCCGCCCAGTCCTT
CATCGAGCGCATGACCAACTTCGACAAGAACCTCCCCAACGAGAAGGTCCTCCCCAA
GCACTCCCTCCTCTACGAGTACTTCACGGTCTACAACGAGCTCACCAAGGTCAAGTAC
GTCACCGAGGGTATGCGCAAGCCTGCCTTCCTCTCCGGCGAGCAGAAGAAGGCTATCG
TTGACCTCCTCTTCAAGACCAACCGCAAGGTCACCGTCAAGCAGCTCAAGGAGGACT
ACTTCAAGAAGATCGAGTGCTTCGACTCCGTCGAGATCAGCGGCGTTGAGGACCGTTT
CAACGCTTCTCTCGGTACCTACCACGATCTCCTCAAGATCATCAAGGACAAGGACTTCC
TCGACAACGAGGAGAACGAGGACATCCTCGAGGACATCGTCCTCACTCTTACTCTCTT
CGAGGATAGGGAGATGATCGAGGAGAGGCTCAAGACTTACGCTCATCTCTTCGATGAC
AAGGTTATGAAGCAGCTCAAGCGTCGCCGTTACACCGGTTGGGGTAGGCTCTCCCGCA
AGCTCATCAACGGTATCAGGGATAAGCAGAGCGGCAAGACTATCCTCGACTTCCTCAA
GTCTGATGGTTTCGCTAACAGGAACTTCATGCAGCTCATCCACGATGACTCTCTTACCT

TCAAGGAGGATATTCAGAAGGCTCAGGTGTCCGGTCAGGGCGACTCTCTCCACGAGCA
CATTGCTAACCTTGCTGGTTCCCCTGCTATCAAGAAGGGCATCCTTCAGACTGTTAAGG
TTGTCGATGAGCTTGTCAAGGTTATGGGTCGTCACAAGCCTGAGAACATCGTCATCGA
GATGGCTCGTGAGAACCAGACTACCCAGAAGGGTCAGAAGAACTCGAGGGAGCGCAT
GAAGAGGATTGAGGAGGGTATCAAGGAGCTTGGTTCTCAGATCCTTAAGGAGCACCCT
GTCGAGAACACCCAGCTCCAGAACGAGAAGCTCTACCTCTACTACCTCCAGAACGGTA
GGGATATGTACGTTGACCAGGAGCTCGACATCAACAGGCTTTCTGACTACGACGTCGA
CCACATTGTTCCTCAGTCTTTCCTTAAGGATGACTCCATCGACAACAAGGTCCTCACGA
GGTCCGACAAGAACAGGGGTAAGTCGGACAACGTCCCTTCCGAGGAGGTTGTCAAGA
AGATGAAGAACTACTGGAGGCAGCTTCTCAACGCTAAGCTCATTACCCAGAGGAAGTT
CGACAACCTCACGAAGGCTGAGAGGGGTGGCCTTTCCGAGCTTGACAAGGCTGGTTT
CATCAAGAGGCAGCTTGTTGAGACGAGGCAGATTACCAAGCACGTTGCTCAGATCCTC
GATTCTAGGATGAACACCAAGTACGACGAGAACGACAAGCTCATCCGCGAGGTCAAG
GTGATCACCCTCAAGTCCAAGCTCGTCTCCGACTTCCGCAAGGACTTCCAGTTCTACA
AGGTCCGCGAGATCAACAACTACCACCACGCTCACGATGCTTACCTTAACGCTGTCGT
TGGTACCGCTCTTATCAAGAAGTACCCTAAGCTTGAGTCCGAGTTCGTCTACGGTGACT
ACAAGGTCTACGACGTTCGTAAGATGATCGCCAAGTCCGAGCAGGAGATCGGCAAGG
CCACCGCCAAGTACTTCTTCTACTCCAACATCATGAACTTCTTCAAGACCGAGATCACC
CTCGCCAACGGCGAGATCCGCAAGCGCCCTCTTATCGAGACGAACGGTGAGACTGGT
GAGATCGTTTGGGACAAGGGTCGCGACTTCGCTACTGTTCGCAAGGTCCTTTCTATGCC
TCAGGTTAACATCGTCAAGAAGACCGAGGTCCAGACCGGTGGCTTCTCCAAGGAGTCT
ATCCTTCCAAAGAGAAACTCGGACAAGCTCATCGCTAGGAAGAAGGATTGGGACCCTA
AGAAGTACGGTGGTTTCGACTCCCCTACTGTCGCCTACTCCGTCCTCGTGGTCGCCAA
GGTGGAGAAGGGTAAGTCGAAGAAGCTCAAGTCCGTCAAGGAGCTCCTCGGCATCAC
CATCATGGAGCGCTCCTCCTTCGAGAAGAACCCGATCGACTTCCTCGAGGCCAAGGGC
TACAAGGAGGTCAAGAAGGACCTCATCATCAAGCTCCCCAAGTACTCTCTTTTCGAGC
TCGAGAACGGTCGTAAGAGGATGCTGGCTTCCGCTGGTGAGCTCCAGAAGGGTAACG
AGCTTGCTCTTCCTTCCAAGTACGTGAACTTCCTCTACCTCGCCTCCCACTACGAGAAG
CTCAAGGGTTCCCCTGAGGATAACGAGCAGAAGCAGCTCTTCGTGGAGCAGCACAAG
CACTACCTCGACGAGATCATCGAGCAGATCTCCGAGTTCTCCAAGCGCGTCATCCTCGC
TGACGCTAACCTCGACAAGGTCCTCTCCGCCTACAACAAGCACCGCGACAAGCCCATC
CGCGAGCAGGCCGAGAACATCATCCACCTCTTCACGCTCACGAACCTCGGCGCCCCTG
CTGCTTTCAAGTACTTCGACACCACCATCGACAGGAAGCGTTACACGTCCACCAAGGA
GGTTCTCGACGCTACTCTCATCCACCAGTCCATCACCGGTCTTTACGAGACTCGTATCG
ACCTTTCCCAGCTTGGTGGTGATAGCAGGGCCGACcccaagaagaagcgtaaggtcGGCGGTGGC
TCGACTAGTTAAATGCCAGATAGAGCTGGATCCCTGCAGGAATTTCCCCGATCGTTCAA
ACATTTGGCAATAAAGTTTCTTAAGATTGAATCCTGTTGCCGGTCTTGCGATGATTATCA
TATAATTTCTGTTGAATTACGTTAAGCATGTAATAATTAACATGTAATGCATGACGTTATT
TATGAGATGGGTTTTTATGATTAGAGTCCCGCAATTATACATTTAATACGCGATAGAAAA
CAAAATATAGCGCGCAAACTAGGATAAATTATCGCGCGCGGTGTCATCTATGTTACTAGA
TC-3′

(B)

5′-TGAGACTTTTCAACAAAGGGTAATATCCGGAAACCTCCTCGGATTCCATTGCCCAGC

TATCTGTCACTTTATTGTGAAGATAGTGGAAAAGGAAGGTGGCTCCTACAAATGCCATC
ATTGCGATAAAGGAAAGGCCATCGTTGAAGATGCCTCTGCCGACAGTGGTCCCAAAGA
TGGACCCCCACCCACGAGGAGCATCGTGGAAAAAGAAGACGTTCCAACCACGTCTTC
AAAGCAAGTGGATTGATGTGATATCTCCACTGACGTAAGGGATGACGCACAATCCCACT
ATCCTTCGCAAGACCCTTCCTCTATATAAGGAAGTTCATTTCATTTGGAGAGAACACGG
GGGACTCTAGATTAATTAAGcctaagaagaagcggaaggttGGTGTGCCTGCGATGGACAAGAAGT
ACTCCATCGGCCTCGACATCGGCACCAACAGCGTCGGCTGGGCGGTGATCACCGACGA
GTACAAGGTCCCGTCCAAGAAGTTCAAGGTCCTGGGCAACACCGACCGCCACTCCATC
AAGAAGAACCTCATCGGCGCCCTCCTCTTCGACTCCGGCGAGACGGCGGAGGCGACC
CGCCTCAAGCGCACCGCCCGCCGCCGCTACACCCGCCGCAAGAACCGCATCTGCTACC
TCCAGGAGATCTTCTCCAACGAGATGGCGAAGGTCGACGACTCCTTCTTCCACCGCCT
CGAGGAGTCCTTCCTCGTGGAGGAGGACAAGAAGCACGAGCGCCACCCCATCTTCGG
CAACATCGTCGACGAGGTCGCCTACCACGAGAAGTACCCCACTATCTACCACCTTCGTA
AGAAGCTTGTTGACTCTACTGATAAGGCTGATCTTCGTCTCATCTACCTTGCTCTCGCTC
ACATGATCAAGTTCCGTGGTCACTTCCTTATCGAGGGTGACCTTAACCCTGATAACTCC
GACGTGGACAAGCTCTTCATCCAGCTCGTCCAGACCTACAACCAGCTCTTCGAGGAGA
ACCCTATCAACGCTTCCGGTGTCGACGCTAAGGCGATCCTTTCCGCTAGGCTCTCCAAG
TCCAGGCGTCTCGAGAACCTCATCGCCCAGCTCCCTGGTGAGAAGAAGAACGGTCTTT
TCGGTAACCTCATCGCTCTCTCCCTCGGTCTGACCCCTAACTTCAAGTCCAACTTCGAC
CTCGCTGAGGACGCTAAGCTTCAGCTCTCCAAGGATACCTACGACGATGATCTCGACA
ACCTCCTCGCTCAGATTGGAGATCAGTACGCTGATCTCTTCCTTGCTGCTAAGAACCTC
TCCGATGCTATCCTCCTTTCGGATATCCTTAGGGTTAACACTGAGATCACTAAGGCTCCT
CTTTCTGCTTCCATGATCAAGCGCTACGACGAGCACCACCAGGACCTCACCCTCCTCA
AGGCTCTTGTTCGTCAGCAGCTCCCCGAGAAGTACAAGGAGATCTTCTTCGACCAGTC
CAAGAACGGCTACGCCGGTTACATTGACGGTGGAGCTAGCCAGGAGGAGTTCTACAA
GTTCATCAAGCCAATCCTTGAGAAGATGGATGGTACTGAGGAGCTTCTCGTTAAGCTTA
ACCGTGAGGACCTCCTTAGGAAGCAGAGGACTTTCGATAACGGCTCTATCCCTCACCA
GATCCACCTTGGTGAGCTTCACGCCATCCTTCGTAGGCAGGAGGACTTCTACCCTTTCC
TCAAGGACAACCGTGAGAAGATCGAGAAGATCCTTACTTTCCGTATTCCTTACTACGTT
GGTCCTCTTGCTCGTGGTAACTCCCGTTTCGCTTGGATGACTAGGAAGTCCGAGGAGA
CTATCACCCCTTGGAACTTCGAGGAGGTTGTTGACAAGGGTGCTTCCGCCCAGTCCTT
CATCGAGCGCATGACCAACTTCGACAAGAACCTCCCCAACGAGAAGGTCCTCCCCAA
GCACTCCCTCCTCTACGAGTACTTCACGGTCTACAACGAGCTCACCAAGGTCAAGTAC
GTCACCGAGGGTATGCGCAAGCCTGCCTTCCTCTCCGGCGAGCAGAAGAAGGCTATCG
TTGACCTCCTCTTCAAGACCAACCGCAAGGTCACCGTCAAGCAGCTCAAGGAGGACT
ACTTCAAGAAGATCGAGTGCTTCGACTCCGTCGAGATCAGCGGCGTTGAGGACCGTTT
CAACGCTTCTCTCGGTACCTACCACGATCTCCTCAAGATCATCAAGGACAAGGACTTCC
TCGACAACGAGGAGAACGAGGACATCCTCGAGGACATCGTCCTCACTCTTACTCTCTT
CGAGGATAGGGAGATGATCGAGGAGAGGCTCAAGACTTACGCTCATCTCTTCGATGAC
AAGGTTATGAAGCAGCTCAAGCGTCGCCGTTACACCGGTTGGGGTAGGCTCTCCCGCA
AGCTCATCAACGGTATCAGGGATAAGCAGAGCGGCAAGACTATCCTCGACTTCCTCAA
GTCTGATGGTTTCGCTAACAGGAACTTCATGCAGCTCATCCACGATGACTCTCTTACCT
TCAAGGAGGATATTCAGAAGGCTCAGGTGTCCGGTCAGGGCGACTCTCTCCACGAGCA
CATTGCTAACCTTGCTGGTTCCCCTGCTATCAAGAAGGGCATCCTTCAGACTGTTAAGG

TTGTCGATGAGCTTGTCAAGGTTATGGGTCGTCACAAGCCTGAGAACATCGTCATCGA
GATGGCTCGTGAGAACCAGACTACCCAGAAGGGTCAGAAGAACTCGAGGGAGCGCAT
GAAGAGGATTGAGGAGGGTATCAAGGAGCTTGGTTCTCAGATCCTTAAGGAGCACCCT
GTCGAGAACACCCAGCTCCAGAACGAGAAGCTCTACCTCTACTACCTCCAGAACGGTA
GGGATATGTACGTTGACCAGGAGCTCGACATCAACAGGCTTTCTGACTACGACGTCGA
CCACATTGTTCCTCAGTCTTTCCTTAAGGATGACTCCATCGACAACAAGGTCCTCACGA
GGTCCGACAAGAACAGGGGTAAGTCGGACAACGTCCCTTCCGAGGAGGTTGTCAAGA
AGATGAAGAACTACTGGAGGCAGCTTCTCAACGCTAAGCTCATTACCCAGAGGAAGTT
CGACAACCTCACGAAGGCTGAGAGGGGTGGCCTTTCCGAGCTTGACAAGGCTGGTTT
CATCAAGAGGCAGCTTGTTGAGACGAGGCAGATTACCAAGCACGTTGCTCAGATCCTC
GATTCTAGGATGAACACCAAGTACGACGAGAACGACAAGCTCATCCGCGAGGTCAAG
GTGATCACCCTCAAGTCCAAGCTCGTCTCCGACTTCCGCAAGGACTTCCAGTTCTACA
AGGTCCGCGAGATCAACAACTACCACCACGCTCACGATGCTTACCTTAACGCTGTCGT
TGGTACCGCTCTTATCAAGAAGTACCCTAAGCTTGAGTCCGAGTTCGTCTACGGTGACT
ACAAGGTCTACGACGTTCGTAAGATGATCGCCAAGTCCGAGCAGGAGATCGGCAAGG
CCACCGCCAAGTACTTCTTCTACTCCAACATCATGAACTTCTTCAAGACCGAGATCACC
CTCGCCAACGGCGAGATCCGCAAGCGCCCTCTTATCGAGACGAACGGTGAGACTGGT
GAGATCGTTTGGGACAAGGGTCGCGACTTCGCTACTGTTCGCAAGGTCCTTTCTATGCC
TCAGGTTAACATCGTCAAGAAGACCGAGGTCCAGACCGGTGGCTTCTCCAAGGAGTCT
ATCCTTCCAAAGAGAAACTCGGACAAGCTCATCGCTAGGAAGAAGGATTGGGACCCTA
AGAAGTACGGTGGTTTCGACTCCCCTACTGTCGCCTACTCCGTCCTCGTGGTCGCCAA
GGTGGAGAAGGGTAAGTCGAAGAAGCTCAAGTCCGTCAAGGAGCTCCTCGGCATCAC
CATCATGGAGCGCTCCTCCTTCGAGAAGAACCCGATCGACTTCCTCGAGGCCAAGGGC
TACAAGGAGGTCAAGAAGGACCTCATCATCAAGCTCCCCAAGTACTCTCTTTTCGAGC
TCGAGAACGGTCGTAAGAGGATGCTGGCTTCCGCTGGTGAGCTCCAGAAGGGTAACG
AGCTTGCTCTTCCTTCCAAGTACGTGAACTTCCTCTACCTCGCCTCCCACTACGAGAAG
CTCAAGGGTTCCCCTGAGGATAACGAGCAGAAGCAGCTCTTCGTGGAGCAGCACAAG
CACTACCTCGACGAGATCATCGAGCAGATCTCCGAGTTCTCCAAGCGCGTCATCCTCGC
TGACGCTAACCTCGACAAGGTCCTCTCCGCCTACAACAAGCACCGCGACAAGCCCATC
CGCGAGCAGGCCGAGAACATCATCCACCTCTTCACGCTCACGAACCTCGGCGCCCCTG
CTGCTTTCAAGTACTTCGACACCACCATCGACAGGAAGCGTTACACGTCCACCAAGGA
GGTTCTCGACGCTACTCTCATCCACCAGTCCATCACCGGTCTTTACGAGACTCGTATCG
ACCTTTCCCAGCTTGGTGGTGATAGCAGGGCCGACcccaagaagaagcgtaaggtcGGCGGTGGC
TCGACTAGTATGCCAGATAGAGCTCAAGTTATTATTAGAATTGTGCCAGGAGGAGGAAC
TAAGACTTTGCAACAAATTATTAACCAGCTTGAGTATTTGTCTAGAAAGGGAAAGCTTG
AGTTGCAAAGATCAGCTAGACATCTTGATATTCCAGTGCCACCAGATCAAATTAGAGAA
CTTGCACAATCATGGGTGACTGAAGCAGGAATTTATGATGAATCACAGAGTGATGATGA
TAGGCAACAAGATCTTACAACTCATATTATTGTGAGTTTCCCAGCTGGAACAGATCAAA
CTGCTGCATATGAAGCTTCTAGAGAGTGGGCAGCAGAAATGTTTGGATCAGGATATGG
AGGAGGAAGATATAATTACCTTACAGCTTACCATGTTGATAGAGATCATCCACATCTTCA
TGTGGTTGTTAATAGAAGAGAATTGCTTGGACATGGATGGCTTAAGATTTCAAGAAGAC
ATCCACAATTGAATTACGATGGACTTAGAAAAAAGATGGCTGAAATTTCTCTTAGACAT
GGAATTGTTCTTGATGCTACTTCTAGAGCTGAAAGAGGAATTGCTGAAAGACCAATTAC
TTATGCTGAACATAGAAGACTTGAAAGAATGCAAGCTCAAAAGATTCAATTTGAAGAT

ACTGATTTTGATGAAACTTCTCCAGAAGAAGATAGAAGAGATCTTTCTCAATCTTTTGA
TCCATTTAGATCTGATCCATCTACTGGAGAACCAGATAGAGCTACTAGACATGATAAGC
AACCACTTGAACAACATGCTAGATTTCAAGAATCTGCTGGATCTTCTATTAAGGCTGAT
GCTAGAATTAGAGTTTCTCTTGAATCTGAAAGATCTGCTCAACCATCTGCTTCTAAGATT
CCAGTTATTGGACATTTTGGAATTGAAACTTCTTATGTTGCTGAAGCTTCTGTTAGAAA
GAGATCTGGAATTTTTGGAACTTCTAGACCAGTTACTGATGTTGCTATGCATACTGTTAA
GAGACAACAAAGATCTAAGAGAAGAAATGATGAAGAAGCTGGACCATCTGGAGCTAA
TAGAAAGGGACTTAAGGCTGCTCAAGTTGATTCTGAAGCTAATGTTGGAGAACAAGAT
ACTAGAGATGATTCTAATAAGGCTGCTGATCCAGTTTCTGCTTCTATTGGAACTGAACA
ACCAGAAGCTTCTCCAAAGAGACCAAGAGATAGACATGATGGAGAACTTGGAGGAAG
AAAGAGAGCTAGAGGAAATAGAAGAGATGATGGAAGAGGAGGAACTTAAGGATCCCT
GCAGGAATTTCCCCGATCGTTCAAACATTTGGCAATAAAGTTTCTTAAGATTGAATCCT
GTTGCCGGTCTTGCGATGATTATCATATAATTTCTGTTGAATTACGTTAAGCATGTAATAA
TTAACATGTAATGCATGACGTTATTTATGAGATGGGTTTTTATGATTAGAGTCCCGCAATT
ATACATTTAATACGCGATAGAAAACAAAATATAGCGCGCAAACTAGGATAAATTATCGC
GCGCGGTGTCATCTATGTTACTAGATC-3′

Supplemental Dataset 2 Complete sequences of the plant codon-optimized *Cas9* and *CvD* expression vectors.

(A) The *Cas9* gene and **(B)** *CvD* fusion gene are both driven the by the cauliflower mosaic virus 35S (CaMV35S) promoter (gray highlight) and the nopaline synthase (NOS) terminator (yellow highlight). The *Cas9* sequence is shown in green and the *virD2* sequence in blue. Red lower case letters indicate the SV40 nuclear localization signal.

(A)

5′-TGAGACTTTTCAACAAAGGGTAATATCCGGAAACCTCCTCGGATTCCATTGCCCAGC
TATCTGTCACTTTATTGTGAAGATAGTGGAAAAGGAAGGTGGCTCCTACAAATGCCATC
ATTGCGATAAAGGAAAGGCCATCGTTGAAGATGCCTCTGCCGACAGTGGTCCCAAAGA
TGGACCCCCACCCACGAGGAGCATCGTGGAAAAAGAAGACGTTCCAACCACGTCTTC
AAAGCAAGTGGATTGATGTGATATCTCCACTGACGTAAGGGATGACGCACAATCCCACT
ATCCTTCGCAAGACCCTTCCTCTATATAAGGAAGTTCATTTCATTTGGAGAGAACACGG
GGGACTCTAGAGGATCCatgtcaaaacatacccgcgtgacttcgtccgaaactgcgatcaaccagcaccgcagcctcaacgtt
gaagggttcaaggtggtttccgcgagactccggtcagccgaatacgagaccttctcgtatcaggcacgcttgctgggactctcggattctatgg
ccattcgcgttgcagtcagaagaatcggaggatttctggagatagacgctcacacgcgcgagaagatggaggcgattcttcagagtatcggc
atattgtcttcgaatgtttcgatgctgctgagcgcgtacgccgaagaccctaggtccgacctggaagcggttagggatgagaggatcgcttttg
gggaggcctttgccgccctcgacggccttcttcgctccatcctctcagtgtccagaagacgcatcgatggctgctcgctgctgaaaggcgctct
ctaaGAGCTCGAATTTCCCCGATCGTTCAAACATTTGGCAATAAAGTTTCTTAAGATTGAA
TCCTGTTGCCGGTCTTGCGATGATTATCATATAATTTCTGTTGAATTACGTTAAGCATGTA
ATAATTAACATGTAATGCATGACGTTATTTATGAGATGGGTTTTTATGATTAGAGTCCCGC
AATTATACATTTAATACGCGATAGAAAACAAAATATAGCGCGCAAACTAGGATAAATTAT
CGCGCGCGGTGTCATCTATGTTACTAGATC-3′

(B)

5′-TGAGACTTTTCAACAAAGGGTAATATCCGGAAACCTCCTCGGATTCCATTGCCCAGC
TATCTGTCACTTTATTGTGAAGATAGTGGAAAAGGAAGGTGGCTCCTACAAATGCCATC
ATTGCGATAAAGGAAAGGCCATCGTTGAAGATGCCTCTGCCGACAGTGGTCCCAAAGA
TGGACCCCCACCCACGAGGAGCATCGTGGAAAAAGAAGACGTTCCAACCACGTCTTC
AAAGCAAGTGGATTGATGTGATATCTCCACTGACGTAAGGGATGACGCACAATCCCACT
ATCCTTCGCAAGACCCTTCCTCTATATAAGGAAGTTCATTTCATTTGGAGAGAACACGG
GGGACGAGCTCGGTACCatggaccttagcggaaacgagaaatcaagaccatggaagaaggcaaacgtcagttcgtctacaa
tttctgatatccaaatgacgaacggcgaaaaccttgagtccggatcgcctacccgtacggaggttctttcaccacgcttggacgacggctctgtt
gactcaagctcctccctttactccggcagcgagcatggcaaccaagcagagatccaaaaggagctgagcgctttgttttcaaacatgagtcttc
cggggaacgatcggagaccggacgagtacatattggtccggcagactgggcaggacgcctttaccggaatcgcgaaaggcaacttggacc
acatgcccacaaaggcagagttcaatgcgtgctgcaggctctacagagatggcgcaggcaattactaccccccgcctctggccttcgataag
atcagtgtgccagctcagctggaggaaacatggggaatgatggaagctaaagaacggaataagttgcgttttcagtacaaattggatgtttgga
atcacgcccacgccgatatggggatcaccggtacggaaattttctatcagacggacaagaacattaagctcgaccgcaattacaagcttcgtc
ctgaggatcgctatgtgcagacagagaggtacggccgccgtgaaattcagaagagatatcagcacgaactccaagcaggcagcctcctgcc
tgatatcatgataaagacgcccaagaacgatatccatttcgtgtaccggttcgccggtgacaactacgcgaacaaacaattctccgagttcgag
cacacagtgaagaggcgctatggtggtgaaactgagatcaagctcaagtccaaatctggcataatgcatgactcaaagtatctggaatcatgg
gagaggggaagcgccgacatccgtttcgccgagtttgttggcgagaaccgcgctcacaatagacagtttccgacagccaccgtgaatatgg
gccaacaacccgatggccagggagggttgaccagagatcgccacgtctcagtggagttcctcatgcagagcgcaccaaactcgccttggg
cgcaggcacttaagaaggggggagctttgggacagggtccaactcttggcccgtgacggtaaccgctaccttagcccccataggctggaata
ctcagacccggagcattttactgagcttatgaaccgggttgggttgccggcatctatgggcagacaatcgcacgctgcctccatcaagtttgaa
aaattcgatgcccaggcggcagttattgtaattaatggtccagaactccgcgatatccacgacctctcacctgaaaacctgcagaacgtcagca
cgaaagacgttatagtcgccgatagaaatgagaacggacagcgcacggggacatatacctccgtcgctgagtacgaaagattgcagttgag
gctgccagcagacgccgcaggtgttctcggagaagctgccgacaagtatagccgtgattttgttcggccggaaccggcttcgagaccgatca
gtgatagtcggaggatctacgagagccggccgagaagtcagagtgttaactcattctgaACTAGTGTCGACCTGCAGagcttt
cgttcgtatcatcggtttcgacaacgttcgtcaagttcaatgcatcagtttcattgcgcacacaccagaatcctactgagtttgagtattatggcatt

gggaaaactgtttttcttgtaccatttgttgtgcttgtaatttactgtgttttttattcggttttcgctatcgaactgtgaaatggaaatggatggagaag
agttaatgaatgatatggtccttttgttcattctcaaattaatattatttgtttttctcttatttgttgtgtgttgaatttgaaattataagagatatgcaaac
attttgttttgagtaaaaatgtgtcaaatcgtggcctctaatgaccgaagttaatatgaggagtaaaacacttgtagttgtaccattatgcttattcact
aggcaacaaatatattttcagacctagaaaagctgcaaatgttactgaatacaagtatgtcctcttgtgttttagacatttatgaactttcctttatgta
attttccagaatccttgtcagattctaatcattgctttataattatagttatactcatggatttgtagttgagtatgaaaatatttttttaatgcattttatgact
tgccaattgattgacaacatgcatcaa-3′

(C)

5′-GAATTCTGAGACTTTTCAACAAAGGGTAATATCCGGAAACCTCCTCGGATTCCATTG
CCCAGCTATCTGTCACTTTATTGTGAAGATAGTGGAAAAGGAAGGTGGCTCCTACAAAT
GCCATCATTGCGATAAAGGAAAGGCCATCGTTGAAGATGCCTCTGCCGACAGTGGTCC
CAAAGATGGACCCCCACCCACGAGGAGCATCGTGGAAAAAGAAGACGTTCCAACCAC
GTCTTCAAAGCAAGTGGATTGATGTGATATCTCCACTGACGTAAGGGATGACGCACAAT
CCCACTATCCTTCGCAAGACCCTTCCTCTATATAAGGAAGTTCATTTCATTTGGAGAGA
ACACGGGGGACTCTAGAGGATCCatgtcaaaacatacccgcgtgacttcgtccgaaactgcgatcaaccagcaccgca
gcctcaacgttgaagggttcaaggtggtttccgcgagactccggtcagccgaatacgagaccttctcgtatcaggcacgcttgctgggactct
cggattctatggccattcgcgttgcagtcagaagaatcggaggatttctggagatagacgctcacacgcgcgagaagatggaggcgattcttc
agagtatcggcatattgtcttcgaatgtttcgatgctgctgagcgcgtacgccgaagaccctaggtccgacctggaagcggttagggatgaga
ggatcgcttttggggaggcctttgccgccctcgacggccttcttcgctccatcctctcagtgtccagaagacgcatcgatggctgctcgctgctg
aaaggcgctctctaaGAGCTCGAATTTCCCCGATCGTTCAAACATTTGGCAATAAAGTTTCTTAA
GATTGAATCCTGTTGCCGGTCTTGCGATGATTATCATATAATTTCTGTTGAATTACGTTAA
GCATGTAATAATTAACATGTAATGCATGACGTTATTTATGAGATGGGTTTTTATGATTAGA
GTCCCGCAATTATACATTTAATACGCGATAGAAAACAAAATATAGCGCGCAAACTAGGA
TAAATTATCGCGCGCGGTGTCATCTATGTTACTAGATCGAATTCGGTCCCCAGATTAGCC
TTTTCAATTTCAGAAAGAATGCTAACCCACAGATGGTTAGAGAGGCTTACGCAGCAGG
TCTCATCAAGACGATCTACCCGAGCAATAATCTCCAGGAAATCAAATACCTTCCCAAGA
AGGTTAAAGATGCAGTCAAAAGATTCAGGACTAACTGCATCAAGAACACAGAGAAAG
ATATATTTCTCAAGATCAGAAGTACTATTCCAGTATGGACGATTCAAGGCTTGCTTCACA
AACCAAGGCAAGTAATAGAGATTGGAGTCTCTAAAAAGGTAGTTCCCACTGAATCAAA
GGCCATGGAGTCAAAGATTCAAATAGAGGACCTAACAGAACTCGCCGTAAAGACTGG
CGAACAGTTCATACAGAGTCTCTTACGACTCAATGACAAGAAGAAAATCTTCGTCAAC
ATGGTGGAGCACGACACACTTGTCTACTCCAAAAATATCAAAGATACAGTCTCAGAAG
ACCAAAGGGCAATTGAGACTTTTCAACAAAGGGTAATATCCGGAAACCTCCTCGGATT
CCATTGCCCAGCTATCTGTCACTTTATTGTGAAGATAGTGGAAAAGGAAGGTGGCTCCT
ACAAATGCCATCATTGCGATAAAGGAAAGGCCATCGTTGAAGATGCCTCTGCCGACAG
TGGTCCCAAAGATGGACCCCCACCCACGAGGAGCATCGTGGAAAAAGAAGACGTTCC
AACCACGTCTTCAAAGCAAGTGGATTGATGTGATATCTCCACTGACGTAAGGGATGAC
GCACAATCCCACTATCCTTCGCAAGACCCTTCCTCTATATAAGGAAGTTCATTTCATTTG
GAGAGAACACGGGGGACGAGCTCGGTACCatggaccttagcggaaacgagaaatcaagaccatggaagaagg
caaacgtcagttcgtctacaatttctgatatccaaatgacgaacggcgaaaaccttgagtccggatcgcctacccgtacggaggttctttcacca
cgcttggacgacggctctgttgactcaagctcctccctttactccggcagcgagcatggcaaccaagcagagatccaaaaggagctgagcg
ctttgttttcaaacatgagtcttccggggaacgatcggagaccggacgagtacatattggtccggcagactgggcaggacgcctttaccggaa
tcgcgaaaggcaacttggaccacatgcccacaaaggcagagttcaatgcgtgctgcaggctctacagagatggcgcaggcaattactaccc
cccgcctctggccttcgataagatcagtgtgccagctcagctggaggaaacatggggaatgatggaagctaaagaacggaataagttgcgttt

tcagtacaaattggatgtttggaatcacgcccacgccgatatggggatcaccggtacggaaattttctatcagacggacaagaacattaagctc
gaccgcaattacaagcttcgtcctgaggatcgctatgtgcagacagagaggtacggccgccgtgaaattcagaagagatatcagcacgaact
ccaagcaggcagcctcctgcctgatatcatgataaagacgcccaagaacgatatccatttcgtgtaccggttcgccggtgacaactacgcgaa
caaacaattctccgagttcgagcacacagtgaagaggcgctatggtggtgaaactgagatcaagctcaagtccaaatctggcataatgcatga
ctcaaagtatctggaatcatgggagaggggaagcgccgacatccgtttcgccgagtttgttggcgagaaccgcgctcacaatagacagtttcc
gacagccaccgtgaatatgggccaacaacccgatggccagggagggttgaccagagatcgccacgtctcagtggagttcctcatgcagag
cgcaccaaactcgccttgggcgcaggcacttaagaaggggagctttgggacagggtccaactcttggcccgtgacggtaaccgctacctt
agcccccataggctggaatactcagacccggagcattttactgagcttatgaaccgggttgggttgccggcatctatgggcagacaatcgcac
gctgcctccatcaagtttgaaaaattcgatgcccaggcggcagttattgtaattaatggtccagaactccgcgatatccacgacctctcacctga
aaacctgcagaacgtcagcacgaaagacgttatagtcgccgatagaaatgagaacggacagcgcacggggacatatacctccgtcgctga
gtacgaaagattgcagttgaggctgccagcagacgccgcaggtgttctcggagaagctgccgacaagtatagccgtgattttgttcggccgg
aaccggcttcgagaccgatcagtgatagtcggaggatctacgagagccggccgagaagtcagagtgttaactcattctgaACTAGTGT
CGACCTGCAGagctttcgttcgtatcatcggtttcgacaacgttcgtcaagttcaatgcatcagtttcattgcgcacacaccagaatccta
ctgagtttgagtattatggcattgggaaaactgtttttcttgtaccatttgttgtgcttgtaatttactgtgtttttattcggttttcgctatcgaactgtga
aatggaaatggatggagaagagttaatgaatgatatggtccttttgttcattctcaaattaatattatttgttttttctcttatttgttgtgtgttgaatttga
aattataagagatatgcaaacattttgttttgagtaaaaatgtgtcaaatcgtggcctctaatgaccgaagttaatatgaggagtaaaacacttgta
gttgtaccattatgcttattcactaggcaacaaatatattttcagacctagaaaagctgcaaatgttactgaatacaagtatgtcctcttgtgttttaga
catttatgaactttcctttatgtaattttccagaatccttgtcagattctaatcattgctttataattatagttatactcatggatttgtagttgagtatgaaa
atattttttaatgcattttatgacttgccaattgattgacaacatgcatcaa-3′

Supplemental Dataset 3 Complete sequences of the plant codon-optimized *virD1* and *virE2* expression vectors.

(A) The *virD1* sequence (green) is driven by the CaMV35S promoter (gray highlight) and the NOS terminator (yellow highlight). **(B)** The *virE2* sequence (blue) is also driven by the CaMV35S promoter (gray highlight) and the E9 terminator is used in this construct (yellow highlight). **(C)** The sequence of *virD1* and *virE2* constructs were also placed in tandem within a single vector with *Eco*RI site (red highlight).

Table S1. The primers used in this study

Primer name	Primer sequence (5′-3′)	Experiments
35s	GACGCACAATCCCACTATCC	multi-nest PCR assay
E9T	ACTGATGCATTGAACTTGACG	multi-nest PCR assay
F1	AGGAAGTTCATTTCATTTGGAG	multi-nest PCR assay
R1	CCCAGCAGCTGTTACAAACT	multi-nest PCR assay
F2	GAGCTCGGTACCATGAGTA	multi-nest PCR assay
R2	GTTGATAATGGTCTGCTAGTTG	multi-nest PCR assay
GFP-Hi-F	ggagtgagtacggtgtgcTTGCACTACTGGAAAAC	deep amplicon sequencing
GFP-Hi-R	gagttggatgctggatggCATGGCGCTCTTGAAGA	deep amplicon sequencing
sgGFP-F	GATTACGCCAAGCTTCCCGGGtggaatcggcagcaa	expression cassette construction
sgGFP-R	TACTGGAATAGTACTCCCGGGccatccactccaagc	expression cassette construction
p35s-F	CCACTGAATCAAAGGCCATGGAGTCAAAGATTCAAA	vector construction
p35s-R	CGCTTCTTCTTAGGCTTAATTAATCTAGAGTCCCCCGT	vector construction
Cas9-F	ACACGGGGGACTCTAGATTAATTAAGCCTAAGAAGAAGCG	vector construction
Cas9-R	TTCGAGCTCGGATCCACTAGTCGAGCCACCGCCGAC	vector construction
VirE2-F	ACGGGGGACGAGCTCGGTACCatggatctttctggcaatgag	vector construction
VirE2-R	GCTCTGCAGGTCGACACTAGTtcaaaagctgttgacgctttg	vector construction
VirD1-F1	ACGGGGGACTCTAGAGGATCCatgtcaaaacacacc	vector construction
VirD1-R1	CGATCGGGGAAATTCGAGCTCctacaaggcacctttc	vector construction
VirD1-F2	AAAACGACGGCCAGTGAATTCAAGCTTGCATGCCTG	vector construction
VirD1-R2	GCTAATCTGGGGACCGAATTCCCGATCTAGTAACAT	vector construction
GFPm-F1	ACGGGGGACGAGCTCGGTACCATGAGTAAAGGAGAA	vector construction

GFPm-R1	GAAGAAGTCGTGCCGCTTGAAAAGCATTGAACACCA	vector construction
GFPm-F2	TCAATGCTTTTCAAGCGGCACGACTTCTTCAAGAGC	vector construction
GFPm-R2	GAACTATACAAATAAACTAGTGTCGACCTGCAGAGC	vector construction
PBI121-GFPmF	GAGAACACGGGGGACTCTAGAATGAGTAAAGGAGAAGAAC	vector construction
PBI121-GFPmR	CATGGCATGGATGAACTATACAAAGGATCCCCGGGTGGTCAGTCC	vector construction
β-Actin-F	GTGTTAGCCACACTGTCCCA	transgenic plant detection
β-Actin-R	CATAGTCGAACCGCCACTGA	transgenic plant detection
GFPm-dF	CACTACTTTCTGTTATGGTGTTC	transgenic plant detection
GFPm-dR	CCACATGGTCCTTCTTGAGTTTGT	transgenic plant detection
GFP-DRTF	GTTTACCCGCCAATATATCCTGTCAGAACTTTTCACTGGAG	vector construction
GFP-DRTR	TGGCAGGATATATTGTGGTGTAAACGAGTTGTAGTTGTATTCC	vector construction
DRTF(universal)	GTTTACCCGCCAATATATCCTGTCAATGTTACGTCCTGTAG	vector construction
GFP-DRTR	TCCAGTGAAAAGTTCTTTTCGCGATCCAGAC	vector construction
ALS-DRTR	GTAGGCGAACACGTCTTTTCGCGATCCAGAC	vector construction
ALS-RE-F	CGGTCAGGTGCTCGGCGGT	RE-PCR
ALS-RE-R	GCCAGGACGGCCCGAGGAC	RE-PCR
ALS-Hi-F	ggagtgagtacggtgtgcGCGTCAGCGACGTGTTCGCC	deep amplicon sequencing
ALS-Hi-R	gagttggatgctggatggGCCCTGCTCGTGGCGGAAG	deep amplicon sequencing
PDS-DRTR	TTGAACGGTTTGGATCTTTTCGCGATCCAGA	vector construction
PDS-RE-F	TAGGCAACATGTCACTTGGCTCTAGAG	RE-PCR
PDS-RE-R	CTCCACTACAGACTGAGCACAAAGCTTC	RE-PCR
PDS-Hi-F	ggagtgagtacggtgtgcCCAAACCGTTCAATGCTG	deep amplicon sequencing
PDS-Hi-R	gagttggatgctggatggCAGCAGCAATTTCATCAG	deep amplicon sequencing
NRT-DRTR	TGATCGCCGCATGGTCTTTTCGCGATCCAGA	vector construction
NRT-RE-F	CCGTCTAGGGATATTCATATTTGTTG	deep amplicon sequencing
NRT-RE-R	TGAGGAGGATGGAGGCGATGAG	deep amplicon sequencing

NRT-Hi-F	ggagtgagtacggtgtgcACAGGTCGGCGGCGGAGT	deep amplicon sequencing
NRT-Hi-R	gagttggatgctggatggGTCCAGAACATGATGGTGGTCGC	deep amplicon sequencing
GSP1	AACCGTTCAATGCTGGAGTTG	ddPCR
GSP2	ATTTCAGTGTCACTCCGTCCAA	ddPCR
FAM-Q	TCTTTGCTCCTGCAGAG	ddPCR
VIC-Q	CTTTGCTCCCGCGGA	ddPCR
1F	ACTCTTTCCCTACACGACGCTCTTCCGATCTgcttGCGTtggagtgagtacggtgtgc	deep amplicon sequencing
2F	ACTCTTTCCCTACACGACGCTCTTCCGATCTgcttGTAGtggagtgagtacggtgtgc	deep amplicon sequencing
3F	ACTCTTTCCCTACACGACGCTCTTCCGATCTgcttACGCtggagtgagtacggtgtgc	deep amplicon sequencing
4F	ACTCTTTCCCTACACGACGCTCTTCCGATCTgcttCTCGtggagtgagtacggtgtgc	deep amplicon sequencing
5F	ACTCTTTCCCTACACGACGCTCTTCCGATCTgcttGCTCtggagtgagtacggtgtgc	deep amplicon sequencing
6F	ACTCTTTCCCTACACGACGCTCTTCCGATCTgcttAGTCtggagtgagtacggtgtgc	deep amplicon sequencing
7F	ACTCTTTCCCTACACGACGCTCTTCCGATCTgcttCGACtggagtgagtacggtgtgc	deep amplicon sequencing
8F	ACTCTTTCCCTACACGACGCTCTTCCGATCTgcttGATGtggagtgagtacggtgtgc	deep amplicon sequencing
9F	ACTCTTTCCCTACACGACGCTCTTCCGATCTgcttATACtggagtgagtacggtgtgc	deep amplicon sequencing
10F	ACTCTTTCCCTACACGACGCTCTTCCGATCTgcttCACAtggagtgagtacggtgtgc	deep amplicon sequencing
11F	ACTCTTTCCCTACACGACGCTCTTCCGATCTgcttGTGCtggagtgagtacggtgtgc	deep amplicon sequencing
12F	ACTCTTTCCCTACACGACGCTCTTCCGATCTgcttACTAtggagtgagtacggtgtgc	deep amplicon sequencing
13F	ACTCTTTCCCTACACGACGCTCTTCCGATCTgcttCAGCtggagtgagtacggtgtgc	deep amplicon sequencing
14F	ACTCTTTCCCTACACGACGCTCTTCCGATCTgcttTCACtggagtgagtacggtgtgc	deep amplicon sequencing
1R	GACTGGAGTTCAGACGTGTGCTCTTCCGATCTctgtGCGTtgagttggatgctggatgg	deep amplicon sequencing
2R	GACTGGAGTTCAGACGTGTGCTCTTCCGATCTctgtGTAGtgagttggatgctggatgg	deep amplicon sequencing
3R	GACTGGAGTTCAGACGTGTGCTCTTCCGATCTctgtACGCtgagttggatgctggatgg	deep amplicon sequencing
4R	GACTGGAGTTCAGACGTGTGCTCTTCCGATCTctgtCTCGtgagttggatgctggatgg	deep amplicon sequencing
5R	GACTGGAGTTCAGACGTGTGCTCTTCCGATCTctgtGCTCtgagttggatgctggatgg	deep amplicon sequencing
6R	GACTGGAGTTCAGACGTGTGCTCTTCCGATCTctgtAGTCtgagttggatgctggatgg	deep amplicon sequencing

7R	GACTGGAGTTCAGACGTGTGCTCTTCCGATCTctgtCGACtgagttggatgctggatgg	deep amplicon sequencing
8R	GACTGGAGTTCAGACGTGTGCTCTTCCGATCTctgtGATGtgagttggatgctggatgg	deep amplicon sequencing
Off-target-PDS1F	AACACCAAAGTGCACCCTCA	potential off target detected
Off-target-PDS1R	GTTGGGACTGTCCGTTTTGC	potential off target detected
Off-target-PDS2F	CCATGGACTCTGAACCCACC	potential off target detected
Off-target-PDS2R	ACAGATTAAAGGGCCACGCA	potential off target detected
Off-target-PDS3F	TGTTTGGAAGAGGGCTGCAA	potential off target detected
Off-target-PDS3R	TTCGGCTCTGAGCGTTTCTT	potential off target detected
Off-target-PDS4F	GGAAGACGGTGTGGGAATCA	potential off target detected
Off-target-PDS4R	GGGCAACTGCATTGTCCTTC	potential off target detected
Off-target-PDS5F	CCATGGTCCGCTTCGATTCA	potential off target detected
Off-target-PDS5R	GCCTCAACGAGTCAGTCGAA	potential off target detected
Off-target-PDS6F	TCCAGGTAGTGGTCGTCGT	potential off target detected
Off-target-PDS6R	CAAACGATGGATCTGCTGCG	potential off target detected
Off-target-PDS7F	AGCGAATTTCGTCGGGTCTT	potential off target detected
Off-target-PDS7R	GGTGGATGACTTACGGCACA	potential off target detected
Off-target-PDS8F	CCCGGGTACATAAACGGGAC	potential off target detected
Off-target-PDS8R	GGATTTTCGTGGCACGCTTT	potential off target detected
Off-target-PDS9F	AACCGGGAAGCTTGAAGAGG	potential off target detected
Off-target-PDS9R	CGGGGAGCACGCTAATGTAT	potential off target detected
Off-target-PDS10F	TTTAGTTGAGCTTGATGCAGCC	potential off target detected
Off-target-PDS10R	CTGCAGATGATTCTGGAGGTGT	potential off target detected
Off-target-PDS11F	TTAGGTAGCGAACTGCAGGC	potential off target detected
Off-target-PDS11R	GCAGTAACTTAGGCCCCGTT	potential off target detected
Off-target-PDS12F	GGATCAATGGACAGGCGAGG	potential off target detected
Off-target-PDS12R	AACACCACAAGGTTCACCCC	potential off target detected

Off-target-PDS13F	AAGGACGAGGACTCAAGGAGA	potential off target detected
Off-target-PDS13R	AAACGATGCGTTTCGTGCG	potential off target detected
Off-target-PDS14F	CGGATAGCGGCAGTAGTCAG	potential off target detected
Off-target-PDS14R	CGGCGACGAGCTTCTTGAT	potential off target detected
Off-target-PDS15F	GATCTGGGTTTACCTCGCCG	potential off target detected
Off-target-PDS15R	AGGTGAGTGGTGTTTGGTGT	potential off target detected
Off-target-ALS1F	CACACGTGACACAGACGCT	potential off target detected
Off-target-ALS1R	AGGAAGAAGGCCTCGTTGATG	potential off target detected
Off-target-ALS2F	GACATCCTCGTGGAGGCG	potential off target detected
Off-target-ALS2R	CCTTGGGGATGTCGACGAG	potential off target detected
Off-target-ALS3F	AGGTTTCGGCTAGCACTCAG	potential off target detected
Off-target-ALS3R	AACGGGTCAAACCCTAACCC	potential off target detected
Off-target-ALS4F	GACGGAGCTGTTCTCAACGA	potential off target detected
Off-target-ALS4R	GGGTTTTATGGGACGGAGGG	potential off target detected
Off-target-ALS5F	TGACCACACAAGGAACTCCG	potential off target detected
Off-target-ALS5R	CTCGATCTCTGCGTCGAACA	potential off target detected
Off-target-ALS6F	GAAGGCCGGGCAGAAGG	potential off target detected
Off-target-ALS6R	TCACACGGCACATCATGACA	potential off target detected
Off-target-ALS7F	TTTCCAGCAGGAACCAAGCA	potential off target detected
Off-target-ALS7R	GGAACACCTTGCTGGAATGC	potential off target detected
Off-target-ALS8F	CTCACCTCGCCTCACCTCA	potential off target detected
Off-target-ALS8R	CATTGCTACGCATCCGCATT	potential off target detected
Off-target-ALS9F	TCGACATCCTTGCACACTCC	potential off target detected
Off-target-ALS9R	TCAAGCCCTGCTATGTCGTC	potential off target detected
Off-target-ALS10F	CGGACAAGAAGCCCTTGAGA	potential off target detected
Off-target-ALS10R	GTGCTGTGGGTGACTCTTGT	potential off target detected

Off-target-ALS11F	CCGTGTCGTACAGCCTCAG	potential off target detected
Off-target-ALS11R	GAGCATGGTGCTCTCGTACC	potential off target detected
Off-target-ALS12F	GCAAGCCCAAGTATTCCGGT	potential off target detected
Off-target-ALS12R	GCAAGCCCAAGTATTCCGGT	potential off target detected
Off-target-ALS13F	ATGGACGAGACCTCCAACCT	potential off target detected
Off-target-ALS13R	TACAAACAGCGTGCGTTGC	potential off target detected
Off-target-ALS14F	ACCGGATTCGTTCATCAGGTT	potential off target detected
Off-target-ALS14R	GGTTTGACCATTCCCCCTGAT	potential off target detected

Note: barcodes sequences are marked in red.

Table S2. Targets and sgRNA sequences in the *GFPm* gene

Target ID	Target sequence	NNNN-Target-PAM-NNN	Target start	Target end	GC% of Target site	Tm of sgRNA:DNA	Score
1	GGGCACAAATTTTCTGTCAG	TAATGGGCACAAATTTTCTGTCAGTGGAGA	70	89	45.00	65.41	0.14
2	GGTGAAGGTGATGCAACATA	AGAGGGTGAAGGTGATGCAACATACGGAAA	97	116	45.00	63.36	0.50
3	GGTGTTCAATGCTTTTCAAG	TTATGGTGTTCAATGCTTTTCAAGCGGCAC	199	218	40.00	64.51	0.58
4	GGAGAGGACCATCTTCTTCA	TGCAGGAGAGGACCATCTTCTTCAAGGACG	263	282	50.00	72.47	0.15
5	GGAATCGATTTCAAGGAGGA	TAAGGGAATCGATTTCAAGGAGGACGGAAA	360	379	45.00	61.6	0.34

Targets marked in red were selected for this experiment.

Table S3. The HDR ratio of the *GFP* gene following transformation with various combinations of vectors in protoplast

Experiments	GFPm	DRT	Cas9	CvD	VirD1	VirE2	HDR ratio (%)			
							First	Second	Third	Average
I	+	+	-	+	+	+	3.409524	3.370122	3.638889	3.472845
II	+	+	+	-	+	+	0.075916	0.042282	0.080808	0.066335
III	+	+	-	+	-	+	0.578165	0.506329	0.819672	0.634722
IV	+	+	-	+	+	-	0.147533	0.144788	0.319022	0.203781
V	+	+	-	+	-	-	0.152489	0.138972	0.173653	0.155038

Table S4. Transformation with the full CvD components and DRTs with and without linkers, and the corresponding HDR ratios

Experiments	GFPm	CvD	VirD1	VirE2	non-linker DRT	Linker DRT	HDR ratio (%)			
							First	Second	Third	Average
I	+	+	+	+	+	-	2.952816	1.052632	2.671344	2.225597
II	+	+	+	+	-	+	5.681818	3.985507	4.225352	4.630893

Table S5. Endogenous rice target genes and the corresponding sgRNA sequences, diagnostic restriction sites and HDR editing ratios

Target gene	sgRNA	Target sequence	Oligo-F	Oligo-R	PAM
OsALS	sgRNA-*OsALS*	CGCGTCCATGGA GATCCACC	tgcgctgaggctcagCGCGTCCATGGAGATCCA CCgttttagagctagaaatagc	ttctagctctaaaacGGTGGATCTCCATGGACGC Gctgagcctcagcgcagcagc	AGG
OsPDS	sgRNA-*OsPDS*	GTTGGTCTTTGC TCCTGCAG	tgcgctgaggctcagGTTGGTCTTTGCTCCTGCA Ggttttagagctagaaatagc	ttctagctctaaaacCTGCAGGAGCAAAGACCAA Cctgagcctcagcgcagcagc	AGG
OsNRT 1.1B	sgRNA-*OsNRT 1.1B*	CGGCGACGGCG AGCAAGTGG	tgcgctgaggctcagCGGCGACGGCGAGCAAG TGGgttttagagctagaaatagc	ttctagctctaaaacCCACTTGCTCGCCGTCGCC Gctgagcctcagcgcagcagc	AGG

(Column Continue)

Target gene	WT	HDR	CvDT (%)					Cas9T (%)					Folds
			First	Second	Third	Fourth	Average	First	Second	Third	Fourth	Average	
OsALS	*Nco*I	-	0.088403	0.109741	0.07097	0.095761	0.091219	0.004269	0.004060	0.002956	0.003803	0.003772	24.2
OsPDS	*Pst*I	*Sca*II	0.594362	0.597431	0.224969	1.296024	0.678196	0.027802	0.014972	0.011552	0.031198	0.021381	31.7
OsNRT 1.1B	-	*Nco*I	0.042245	0.012454	0.025862	-	0.026854	0.000787	0.001469	0.002845	-	0.001700	15.8

Table S6. *OsPDS* gene HDR ratio in 32 groups of barcodes

HDR ratio (%)	F3	F4	F5	F6
R1	0.525701	0.000000	0.551471	0.410509
R2	0.125549	0.000000	0.475737	0.650936
R3	0.396825	0.416667	0.000000	0.449944
R4	0.273973	0.171821	0.158604	0.920810
R5	0.300481	0.349040	0.530035	0.000000
R6	0.715680	0.000000	0.528302	0.000000
R7	0.146520	0.336134	0.770416	0.000000
R8	0.000000	0.112613	0.41958	0.342466

Table S7. Potential off-target sites of the sgRNAs used in this study

No.	Chrom	Position	Sequence	Off-score	Gene	Region	No. of mismatches	sites sequencing
Potential off target sites of sgRNA targeting *OsPDS*								
1	chr05	14691244	TTTGGTCCTTGGTCCTGCAAAGG	0.417	Os05G0317200	intron	4	No
2	chr02	27883652	GTTGTTCTCTGCACCTGCAGGGG	0.192	Os02G0682200	CDS	3	No
3	chr09	17017254	CTTCGTCATTGCTCATGCAGAGG	0.067	Os09G0453700	3' UTR	4	No
4	chr02	7619495	GTAGTTCTTTGCTCCATCAGAGG	0.049	Os02G0234450	CDS	4	No
5	chr10	19117296	CTTGGTCTTCACTCGTGCAGAGG	0.019		intergenic	4	No
6	chr01	23820858	GTTGGTCAGCGCTCTTGCAGAGG	0.018		intergenic	4	No
7	chr10	14578906	GTTGGTCTTGCCTCCTACAGAGA	0.014		intergenic	4	No
8	chr02	15202309	GGTGGTCTTTTCTCCTGAAGATG	0.007		intergenic	4	No
9	chr03	19736755	GTTGGTCTTGGCCCCTGCATCGC	0.006		intergenic	4	No
10	chr04	1674312	GTTGATCTTTGTTCTTGGAGAGG	0.004		intergenic	4	No
11	chr03	6134301	GGTGTTCTTTTCTCCTGCAGATG	0.004	Os03G0216700	intron	3	No
12	chr02	29739001	GTTGGTTATTGCCCATGCAGAGT	0.002	Os02G0716800	intron	5	No
13	chr04	29510387	GTTGTTCTTTTCTACTGAAGAGA	0.002		intergenic	5	No
14	chr03	33304845	GTTGGTCGGTGCTCGTGCAGATG	0.001	Os03G0799000	intron	4	No
15	chr10	22745365	AGTGGTCTTTGCTCTTGCAGAGT	0.001		intergenic	4	No
Potential off target sites of sgRNA targeting *OsALS*								
1	chr04	19169517	CGCGTCAATGGAGATCCACCAGG	0.75	Os04G0389800	CDS	1	No
2	chr04	19143712	CGCGTCGATGGAGATCCACCAGG	0.471	Os04G0389100	CDS	1	No
3	chr09	726010	CGCGCACATGGAGGTCAACCAGG	0.202		intergenic	4	No
4	chr10	4633219	CGCGTCCAAGGAGTTCCAGCAGG	0.062		intergenic	3	No
5	chr10	4629927	CGCGTCCAAGGAGTTCCAGCAGG	0.062	Os10G0167200	CDS	3	No
6	chr06	29977160	CAAGTCCATGCAGATCCACCACG	0.034	Os06G0708600	CDS	4	No

7	chr03	11254025	CACGTCCAAGAAGATCCACCATG	0.031	Os03G0314450	CDS	4	No
8	chr03	27741076	CGCGTCGATGGAGACCATCCAGG	0.03	Os03G0693000	CDS	4	No
9	chr10	4498364	CGCGTCCAGGGAGTTCAAGCAGG	0.019	Os10G0165300	CDS	4	No
10	chr07	11789879	CACGACCAAGGAGATCCACCATG	0.015	Os07G0299800	CDS	4	No
11	chr12	23093882	CGCGTCCATGGACTTCCACCCGC	0.005	Os12G0563000	CDS	3	No
12	chr09	4571497	CGCGTCCATGGAGCTCGTCAAGG	0.003		intergenic	4	No
13	chr09	19158387	CACGTCCATGGAGGTCGAGCAGG	0.003	Os09G0494500	CDS	3	No
14	chr02	11485221	GGCCTCCATGGAGTTCAACCAGT	0.002	Os02G0299300	3' UTR	3	No

No: not detected.
